# Corrugator activity reflects integrated simulation and multifaceted evaluation when processing affective language: Frowning responses to reading about emotions of morally good and bad characters

**DOI:** 10.3758/s13415-026-01464-8

**Published:** 2026-08-07

**Authors:** Li Kloostra, Marijke Beulen, Jos J. A. van Berkum, Marijn E. Struiksma

**Affiliations:** 1 Language, Literature, and Communication, Institute for Language Sciences, Utrecht University, Utrecht, The Netherlands; 2 Language, Literature, and Communication, Utrecht University, Utrecht, Netherlands

**Keywords:** Affective language processing, Emotion, Embodiment, Facial electromyography, Psycholinguistics

## Abstract

Corrugator supercilii (“frowning muscle”) activity tracks affective valence of linguistic stimuli. According to embodied cognition accounts, this reflects simulation of the emotion at lexical and/or situational levels. Additionally, it may indicate evaluation, i.e., how we feel about an affective event within a context. This study used facial electromyography (fEMG) to examine corrugator responses as participants read short stories. Each story included segments, describing some character’s morally good or bad behavior, a behavior rating, and sentence-initial phrases describing the character’s positive or negative affective states (e.g., “Mark is frustrated”), followed by the reason for this. Our prior studies showed substantially more frowning when good characters were “frustrated” compared with “happy” but minimal differences for bad characters, suggesting a multiple-drivers account where both simulation and evaluation influence responses. Crucially, they can counteract in the context of bad characters, e.g., “happy” has positive valence but is unfair in context and the opposite applies to “frustrated.” To emphasize characters’ moral status and amplify fairness-based evaluations, an embedded morality judgment rating task was introduced. We hypothesized stronger frowning for “happy” than “frustrated” bad characters due to fairness considerations now outweighing simulation. However, results revealed increased frowning for “frustrated” bad characters. The rating task might have unexpectedly reduced, rather than boosted, evaluative processing through cognitive interference. Nevertheless, previously observed frowning patterns for moral characters were replicated. These findings support a multiple-drivers model but suggest that evaluative processes are more complex and multifaceted than current models capture, calling for revisions to incorporate these complexities into affective processing theories.

**Supplementary Information:**

The online version contains supplementary material available at https://doi.org/10.3758/s13415-026-01464-8.

## Introduction

Humans can completely immerse themselves in a good book. Reading about a heartwarming situation where two long lost lovers finally meet each other again can make our hearts flutter, whereas reading about, say, someone frantically trying to find their child in a busy park can make us feel stressed. How does that work? In order to process language, theories of grounded cognition postulate that people mentally simulate the concepts and situations that it refers to (Barsalou, 2008; Hinojosa et al., 2020; Wilson-Mendenhall et al., 2011; Zwaan, 2016). Evidence for simulation has been found for language that refers to perceptual experience (Zwaan & Pecher, 2012; Zwaan et al., 2002), but also for language that refers to, or implies, voluntary motor behavior (Hauk et al., 2004; Willems & Casasanto, 2011). As might be expected, processing language referring to *emotions* or the bodily expressions typically associated with them also involves some sensorimotor simulation, with readers or listeners subtly reinstating part of the emotional state described or implied (Majid, 2012; Niedenthal et al., 2009; Winkielman et al., 2015). For example, research with facial electromyography (fEMG) has revealed that readers or listeners rapidly and automatically contract the *corrugator supercilii* or “frowning muscle” in response to a word like “angry” and actually relax this muscle somewhat in response to a word like “happy” (Foroni & Semin, 2009; Larsen et al., 2003; for review, see Van Berkum et al., 2023).

Such rapid affective simulation not only occurs when reading isolated words but also when reading phrases, sentences, or stories that describe or imply a character’s emotional state (Fino et al., 2016; Niedenthal et al., 2009). This may to some extent reflect the retrieval of individual word meanings (e.g., “Mark,” “is,” “angry”) but can also reflect the construction of, and sustained attending to, a mental model of what is being described (e.g., imagining an angry Mark; Van Berkum et al., 2009; 't Hart et al., 2018, 2019). The language-driven emotion simulation picked up in facial EMG research can therefore already reflect two different processes involved in merely understanding what is being said: lexical retrieval and situation modeling.

Using our emotion systems to simulate what is being said poses an interesting puzzle. After all, the primary function of emotion is not to simulate linguistically conveyed meaning but to *evaluate* how things in the world relate to our interests and to motivate us to act accordingly (Adolphs, 2019; Fischer & Manstead, 2016; Frijda, 2007; Panksepp & Biven, 2012; Scarantino, 2017; Scherer & Moors, 2019; for a review, see Van Berkum, 2022). Emotions, such as anger, fear, happiness, or interest, have evolved to help us cope with the physical and social environment and to respond accordingly when faced with relevant situations. These systems suggest rapid reactions to all kinds of threats and opportunities, including an offensive, scary, interesting, or adorable person. The same systems are also at work when we *read or hear* about such things. For example, reading about an interesting person may arouse curiosity, and reading about a morally despicable person may elicit a feeling of moral indignation (Krumhuber et al., 2018). Both feelings might elicit a facial response. In such linguistically mediated cases, the first role of emotion systems that comes to mind is to assist in rapidly evaluating what is being asserted and implied and to motivate you to act appropriately. At the same time, the way that we employ our emotion systems and subsequently evaluate the morality of a given situation differs from person to person, depending on our personal experiences and beliefs.

The fact that our emotion systems are primarily supposed to evaluate our physical and social environment, including all the language that surrounds us, presents an interesting puzzle: what if we also need those emotion systems to *simulate* linguistically conveyed meaning, as demonstrated by embodied language processing research? And what if—as will quite often be the case—the language-driven simulated emotion is not identical to the *evaluative* emotion we have about what we read or hear? Reading “Mark was happy when he got an expensive watch” might lead us to feel good for Mark, but if we previously read how this character mistreated his dogs, we will probably evaluate Mark’s happiness very differently. In the latter case, Mark’s happiness from getting the watch will most likely be evaluated negatively by readers, because this positivity seems unfair or undeserved given the context of his behavior. This can evoke a sense of moral indignation and subsequentially elicit frowning, despite the adjective’s positive valence. Similarly, reading that “Mark was angry at discovering a dent in his parked car” may elicit a negative evaluation if we like this person “Mark”, whereas it might lead to a more positive evaluation, and perhaps even some *Schadenfreude* (Ouwerkerk et al., 2018; Smith & van Dijk, 2018), if we hate him for mistreating dogs, because justice is now being served. With such involuntary evaluations rapidly affecting our facial expressions (Ekman, 1993; Scarantino, 2017), how does this relate to the subtle “derivative” facial expressions that we make as part of merely *simulating the meaning of* “Mark was angry” or “…happy?”.

### Prior work

In three prior studies ('t Hart et al., 2018, 2019, 2021), we measured corrugator or “frowning muscle” EMG to explore how language-driven simulation of (lexical-semantic or character) emotion interacts with the reader’s own evaluation of what’s being described. We used facial EMG, because it allows us to track language-driven emotion as the language— and the emotion—unfolds over time and in a way that is both technically and inferentially a bit more straightforward than with EEG or MEG. We focused specifically on the corrugator muscle, because, unlike the zygomaticus major associated with smiling, the corrugator robustly picks up on emotional valence *in two directions* (Larsen et al., 2003; Tan et al., 2012; Van Berkum et al., 2023), with negative valence leading to a large increase in muscle activity, and positive valence leading to a decrease, relative to a neutral baseline. Furthermore, with verbal materials, corrugator EMG signals turn out to be somewhat more sensitive to one’s emotional state than zygomaticus EMG signals (Larsen et al., 2003; Van Berkum et al., 2023).

For the first two studies ('t Hart et al., 2018, 2019), participants were presented with short morally loaded narratives in which we first described a character’s moral or immoral behavior (e.g., Mark is helping someone out vs. calling someone names) and then described the character’s affective state (e.g., “Mark is happy” vs. “Mark is angry”). The event that caused them to experience this emotion was reported later in the story. Flipping a character’s moral status allowed us to manipulate the reader’s evaluation of this person’s later affective state as fair (just, deserved) or unfair (unjust, undeserved) and to explore how such fairness-based moral evaluations interacted with language-driven simulation as the affective state adjective was being processed. The critical finding was that, while negative state adjectives (e.g., “angry”) led to more corrugator activity than positive state adjectives (e.g., “happy”) when the character experiencing those states had been introduced as a morally good character, the corrugator EMG response to negative and positive state adjectives did *not* differ when the character had been introduced as an *im*moral character. We replicated the finding in a third study ('t Hart et al., 2021), where we replaced the story context by simply designating a character as “good” or “bad” before immediately describing their emotional response to some event.

Work in other labs, with different materials, has revealed a similar phenomenon. In an EMG study on social unexpectedness, for example, descriptions of moral transgressions generated a larger corrugator response if they were committed by characters previously described in a positive, rather than a negative, way (Bartholow et al., 2001). In an EMG study involving Italian ingroup or outgroup politicians (Fino et al., 2019), the corrugator responded strongly to negative versus positive emotional expression descriptions (e.g., “Berlusconi frowns” versus “Berlusconi smiles”) if the politician belonged to the participant’s political ingroup but not if they belonged to the participant’s political outgroup. In all studies discussed, a negative perception of a character eliminates or strongly attenuates the “normal” adjective valence effect in corrugator EMG, with negative state or action words (e.g., “angry,” “frowns”) no longer eliciting a stronger corrugator response than positive state or action words (e.g., “happy,” “smiles”).

**Fig. 1:**
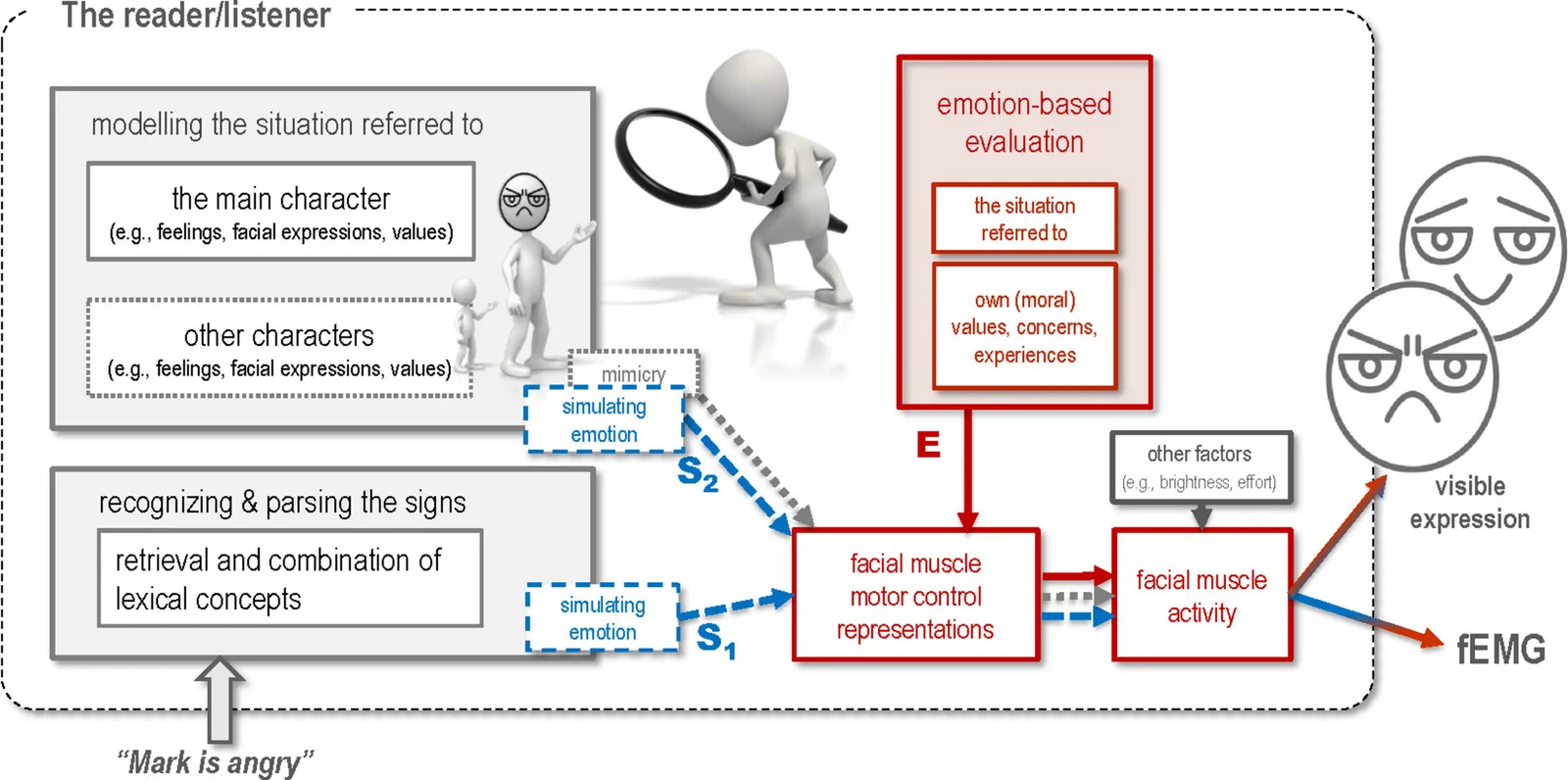
The multiple-drivers account. The multiple-drivers account, adapted from the broader fACL model (Van Berkum et al., 2023), shows how facial muscle activity may be affected by both simulation and evaluation of affective language during processing. Additionally, the model recognizes that there may be other factors, including mimicry, that drive our facial expressions

Because simple models involving *only* language-driven simulation, or *only* moral evaluation, cannot easily explain these results, we converged on a *multiple-drivers* model of corrugator activity during affective language comprehension (ALC) (Fig. 1, adapted from Van Berkum et al., 2023). In this model, the corrugator EMG signal simultaneously reflects both emotion simulation (at the lexical and/or situation model level) *and* emotional evaluation of what is being asserted, i.e., “Mark is happy,” in the moral context.

This multiple-drivers account proposed that in the case of a morally good character, negative emotion induced by simulation at e.g., “Mark is angry” adds up with the negative emotional evaluation of an unfair outcome, and positive emotion induced by simulation at “Mark is happy” adds up with the positive moral evaluation of a fair outcome. The net result is a substantially increased activation of the corrugator at negative emotion words, relative to positive ones. In the case of a morally bad character, however, negative emotion induced by simulation at “Mark is angry” is *counteracted by* the positive emotional evaluation of a fair outcome, and positive emotion induced by simulation at, “Mark is happy” is *counteracted by* the negative emotional evaluation of an unfair outcome. We argue that this may have happened to such an extent that, with our materials, no net valence effect at negative versus positive emotion words remains.

Although a multiple-drivers model sounds plausible, and simultaneous control by multiple systems is a common design feature of the brain, the account is only modestly parsimonious, because it explains a null result as the net effect of two counteracting forces. Independent evidence for the assumed simultaneous control of facial movement by simulation and evaluation, and the net cancelling out of these two forces in the case of our immoral character conditions, would be desirable. In a separate part of the design of our third EMG-study ('t Hart et al., 2021), we therefore tried to reduce the presumed force of evaluation by replacing morally bad characters with members of a much weaker outgroup, defined by the fact that a (fake) personality test profile (labelled “P-type” or “O-type”) differed from that of the reader. The logic here was that, with an attenuated level of positive emotion (e.g., Schadenfreude) when something bad happens to such a minimal outgroup character instead of a morally despicable character, the presumed increase in corrugator activity caused by simulating the meaning of, for example, “Mark is angry” would be *only partially* counteracted by a positive evaluation of the entire situation.

As it turned out, however, the differential corrugator effect to negative versus positive state adjectives (e.g., “angry” vs. “happy”) was as large for minimal outgroup members as it was for minimal ingroup members and simply resembled the standard EMG-findings associated with those two sets of emotion adjectives in neutral contexts. This could be taken to refute the multiple-drivers account, but a more plausible explanation is that when reading fictitious stories about happy or angry people, readers do not really care about whether those people have a different score on some abstract personality test than the readers themselves. In such a situation, the model merely predicts more corrugator activity to negative state adjectives than to positive ones because of (lexical and/or situation-model) simulation, which is perhaps slightly elevated because of general empathy with *both* types of characters in the face of their adversity.

### The current study

In this current study, we took a different approach to testing the multiple-drivers account. Instead of attempting to weaken the force of (group-dependent) moral evaluation to explore the remaining effect of emotion simulation on the corrugator, we now attempted to *boost* it. We did this by adding a secondary task in the form of a morality judgement rating task, embedded within the same morally loaded narratives that were used in the ‘t Hart et al. (2019) study. Table 1 below exemplifies the approach with one of our stimuli.

**Table 1:**
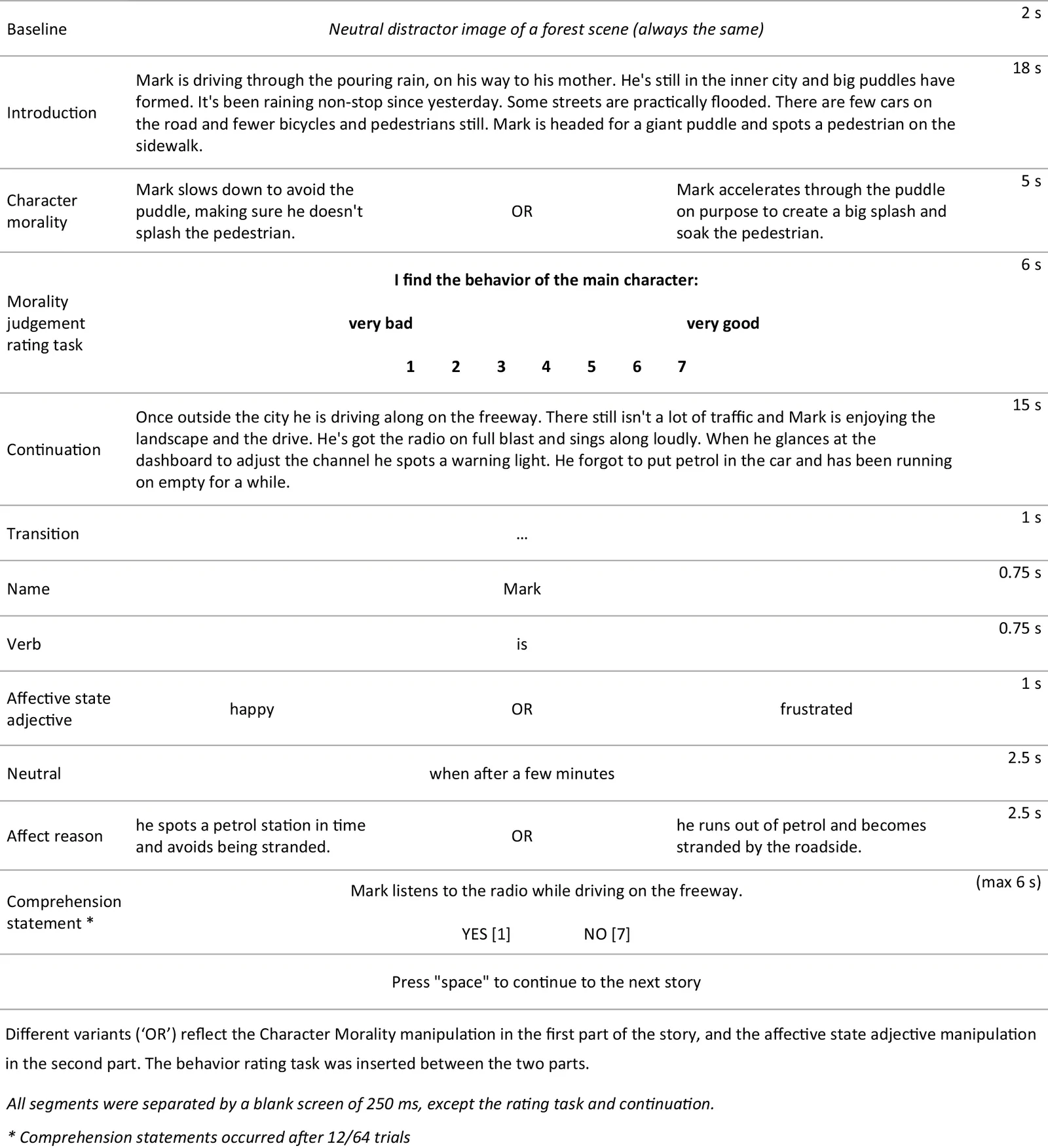
Structure and timing of an example stimulus

Just like in the ‘t Hart et al. (2019) study, each story began with a short introduction in which the scene was set, after which the main character was described to engage in a bit of morally good or bad behavior. Because the items up to this point are identical to prior experiments, we expect to replicate our earlier findings: readers showing substantially increased corrugator activity (i.e., frowning) when reading about immoral behavior, and somewhat decreased corrugator activation (i.e., relaxation) when reading about moral behavior. This can also be seen as a check of the morality manipulation. Rather than simply continuing the story as we did in the ‘t Hart et al. (2019) experiment, readers now had to rate the behavior of the main character on a 7-point scale (“very bad” to “very good”) before the story continued. The critical hypothesis here is that requesting participants to actively reflect on and rate the moral or immoral behavior will increase the salience of the character’s inferred moral status, which should increase fairness-based evaluation as an effect of this moral context (Cannon et al., 2011).

After the morality judgement rating task, the story would continue exactly like in our previous study ('t Hart et al., 2019). Readers first saw a continuation segment that coherently set the stage for the critical sentence (e.g., “Mark is happy/frustrated when…”). This sentence was presented in a piecewise fashion, allowing us to time-lock the EMG signal to its most critical part: the affective state adjective (e.g., “happy” vs. “frustrated”). This adjective is again our critical segment where we expect our results to differ from our previous results: a large differential corrugator effect for negative over positive state adjectives in the case of moral characters, but no such effect for immoral characters. The latter null effect was explained by the assumption that, under the multiple-drivers account, the average effect of emotion simulation is simultaneously *counteracted* by a similar-sized effect of fairness-based moral evaluation (e.g., is it fair/unfair that some bad character feels happy/frustrated).

For our current experiment, however, we hypothesized that an embedded morality judgment rating task would boost fairness-based evaluation while the contribution of emotion simulation remains unchanged. Under the multiple-drivers account, we predicted that, for immoral characters, this would lead to a moderate net *decrease* of corrugator activity at negative adjectives, such as “frustrated’ (some negatively valenced simulation of the word or event but mainly positive evaluation) and a moderate net *increase* at words like “happy” (some positively valenced simulation but mainly negative evaluation). In other words, the net corrugator activity is hypothesized to reflect the opposite of the adjective’s valence. In contrast, for moral characters the two forces are again expected to join, thus aligning with the adjective’s valence. As a result of boosting the evaluation force, the joint effects of simulation and evaluation may be descriptively somewhat larger than observed in earlier studies. In any case, our crucial prediction is that our corrugator EMG waveforms will be flipped depending on the character morality condition: when referring to moral characters, we expect a clear differential pattern with a clear increase (frowning) for negative adjectives compared to positive adjectives. We expect a moderate differential pattern for immoral characters to lie in between these, with somewhat higher activity for positive adjectives than negative adjectives.

In all our stories, the affective state adjective was followed by a specific reason for the affective state experienced by the main character. In our earlier work ('t Hart et al., 2019, 2021), this so-called “affect reason” elicited renewed corrugator activity, generating comparable EMG-patterns as observed at the state adjective. We expect to see a similar thing happening in our current study. However, because the input provided here is distributed over a multiword clause, with the reasons for positive and negative affect usually differing on more than one word, descriptions of affect reasons were much less well-controlled in terms of lexical variables and time-locking precision. As in our earlier work, we make no detailed predictions for this segment.

In sum, we expect corrugator EMG waveforms to show much more frowning activation when morally good characters are described as “frustrated” compared to “happy”. However, for adjectives describing morally bad people we now expect a reversed pattern, i.e., more frowning for "happy" compared with "frustrated." Moreover, as a result of the additive and opposing forces of simulation and evaluation hypothesized for moral and immoral characters respectively, the flipped pattern of EMG waveforms for immoral characters is expected to be more moderate. We expect them to lie in between the substantially different corrugator EMG waveforms for moral characters.

## Materials and methods

### Participants

For the current study, we tested 60 participants (49 females, 10 males, and 1 person identified as neither), with ages ranging from 18 to 29 (*M* = 21.92 year, *SD* = 2.61) who were recruited from the participant database of Utrecht University’s Institute for Language Sciences (formerly Utrecht Institute of Linguistics OTS). All participants were native Dutch speakers and pursuing a university or HBO (applied higher education) degree. Furthermore, they had no Botox injections in the face and normal or corrected-to-normal vision. Those who had participated in any of the previous studies with (variants of) our stimuli or attended lectures or presentations about the purpose of this study were excluded from the participant pool. Participants received a compensation of €20 in cash for their participation in the 2-hour experiment.

This study and its procedures were approved by the Faculty Ethics Assessment Committee – Humanities of Utrecht University (reference number 22–013-02). All participants read the information letter detailing the nature of the experiment and the procedures before the start of the experiment. The informed consent form emphasized that participants have the right to withdraw consent at any time without having to explain why. The consent form was implemented in the coding of the software used to present our stimuli; the experiment only started running if participants pressed the “YES, I consent” button.

### Materials and design

The experiment had a fully crossed, 2 × 2, within-subjects design, crossing Character Morality (moral vs. immoral) and Critical Event Valence (positive vs. negative). Our main dependent variable was activity of the *corrugator supercilii* as indexed by EMG and measured with surface electrodes (further description below). We also measured activity of the *zygomaticus major*; however, this was not included as a dependent variable in our analyses for reasons mentioned below.

We used the same 64 Dutch narrative stimuli as ‘t Hart et al. (2019), which had been pretested for ‘t Hart et al. (2018), but now with the addition of an embedded morality judgment rating task (Table 1). Owing to our design, there were four versions of each narrative. We reused the four already existing pseudo-randomized lists, each contained the 64 narratives in which: 1) per list, each narrative occurred once in one of the four variants; 2) all participants saw 16 narratives in each of the four conditions (8 with a male and 8 with a female main character); 3) average item properties in each list were similar in terms of prosociality and expectedness; 4) two lists were the reversed order of the two other lists; 5) each narrative occurred with both moral and immoral and both female and male main characters across the four different lists, with the exception of nine narratives that had fixed gender due to stereotypical behavioral expectations.^1^

Our newly added embedded task, the morality judgment rating, presented the words “Het gedrag van de hoofdpersoon vind ik:” (i.e., “I find the behavior of the main character”) and a rating scale that horizontally presented the numbers 1 through 7, labelled “heel slecht” (“very bad”) on the left end and “heel goed” (“very good”) on the right. The task was presented for 5.25 ms during which participants had to press one of the seven letters on the bottom row of a QWERTY keyboard, correspondingly labeled from 1 through 7 with stickers. Although the main goal of this embedded rating task was boosting moral evaluation of the later adjective segment, it also functioned as an exploratory self-report measure of our readers’ judgement of the character’s behavior. To encourage participants to carefully read the entire narratives, we also distributed twelve comprehension questions throughout the experiment in the form of statements about the narrative directly preceding it. Every time such a statement was presented, participants had to indicate whether it was true or false by pressing buttons for “Yes” or “No,” and all statements were identical for all four stimuli lists. Six of the 12 statements were true, and the correct answers were not reliant on experimental conditions.

We also included two additional questionnaires for exploratory purposes. In our Story Fairness Questionnaire, we presented our narratives again, now in its entirety, and asked participants to rate how fair they thought the ending of each narrative was on a horizontal 7-point rating scale with the labels “very fair” and “very unfair” at then ends. The Story Fairness Questionnaire aimed to explore conscious fairness-based evaluation of our stimuli and to see to which extent it would be in line with the EMG responses to the state adjectives, as frowning was expected to be modulated by spontaneous or even unconscious evaluation. There were four versions of the Story Fairness Questionnaire corresponding to all four pseudo-randomized stimuli lists used in their EMG session.

Finally, we administered a partly custom made task that we coined the Justice Trait Questionnaire. This task aimed to obtain some insight into moral evaluation and moreover, to explore participants’ sensitivity for and attitude towards justice. The first part consisted of two open questions asking participants for comments on their judgement process during the Story Fairness Questionnaire and on characters and situations in the narratives. The second and main part of the questionnaire was inspired by the Moral Foundations Questionnaire (MFQ) by Graham et al. (2008) and included custom made statements dealing with justice related to outcomes for good and bad people. For example, “If someone behaves badly, they also deserve to be treated badly by others” and “I think that it’s unfair if something good happens to a bad person.” Participants had to rate to which extent they (dis)agreed with them on a 5-point rating scale from “Completely true” to “Completely untrue.”

### Procedure and data acquisition

Participants were welcomed and seated in a comfortable chair where they were asked to read the information letter unless they indicated that they had already read it beforehand. They were seated at approximately 60 cm from the 15″6 laptop screen (1366 × 768 resolution) on which we presented our experimental stimuli and collected participants’ answers indicated by button responses. After participants confirmed that they were properly informed and agreed to participate in the experiment, we started preparations for the fEMG experiment. After participants had read the detailed instructions and performed two practice trials to familiarize themselves with the procedure, they had the opportunity to ask any remaining questions before starting the experimental stimuli.

The stimuli were presented in Times New Roman (font size 20), and segment presentation was automatically timed, using Presentation software (v23.0, NeuroBS), as indicated in Table 1. The presentation rate, i.e., starting the next trial by pressing the space bar, was self-timed. The experimental trials were divided into four roughly equal blocks with three breaks during which we checked how the participant was doing and offered them some water.

Facial EMG studies on affective valence generally include both the *corrugator supercilii* to measure frowning and the *zygomaticus major*, involved in upwards movement of the mouth corners, is often measured as an indication of smiling (Larsen et al., 2003). However, the zygomaticus appears to be a less reliable measure of valence, because it has a relatively poor level of repeatability and it is more sensitive to crosstalk from neighboring muscles associated with negative emotions, for instance disgust (Larsen et al., 2003; Tan et al., 2012). Our main focus is therefore on the corrugator signal, and although we did measure the zygomaticus for baseline selection and comparability reasons ('t Hart et al., 2018, 2019, 2021), findings from the zygomaticus are not reported in this paper. Facial EMG was continuously measured on the right side of the face with reusable Ag/AgCl electrodes (2-mm contact area) over the standard recording sites for the corrugator and zygomaticus (Van Boxtel, 2023). The raw EMG data were recorded with a sampling rate of 2048 Hz by using the NeXus-10 MKII biofeedback device (Mind Media) in combination with BioTrace + software (MindMedia).

After the EMG part was finished, the electrodes were removed, and participants continued with the Story Fairness Questionnaire and Justice Trait Questionnaire on a lab computer followed by a short exit questionnaire. When participants finished the questionnaires, they were debriefed, given their financial compensation, and thanked for their participation. An experiment session lasted approximately 2 hours in total.

### Data preparation and analyses

#### Analysis of fEMG

A detailed explanation of all the steps in the analyses and scripts used can be found in our online OSF repository (https://doi.org/10.17605/OSF.IO/UENMR). We provide a summary of the details here. We used a custom made tool, EDF + Checker (https://github.com/UiL-OTS-labs/EDFPlusChecker), to check for possible time differences between the experiment triggers of the segments and the corresponding markers in the raw EMG signals as these were used for time-locking. Any inconsistencies were corrected. All checked raw data files can be found in our online data repository (https://doi.org/10.24416/UU01-1PAHMB). We then used BrainVision Analyzer 2 (Version 2.2) to filter the checked data with a band-pass filter between 20 and 500 Hz (48 dB/octave roll-off) and a notch filter at 50 Hz (Van Boxtel, 2010) and to perform signal rectification.

For baseline selection, we used BrainVision Analyzer to create a segment containing the 2,000 ms of baseline activity, recorded during the pre-stimulus neutral distractor image of a forest scene, and the first 2,000 ms of activity during the start of the trial. We visually inspected these segments for remaining artifacts and manually selected maximally long epochs of artifact-free baseline signal (i.e., “quiet signal” free of extreme bursts), with a minimum length of 500 ms in at most two windows, for each participant and each narrative. A trial was excluded from analysis if a baseline epoch of 500 ms could not be found (resulting in 2.58% lost trials).

After baseline selection, following our preregistration, the data were segmented into 58-s epochs, each containing a full trial from the start of the baseline until past the end of the affect reason segment, and exported to MatLab (R2019a) for further segmentation into three parts, time-locked to the onsets of the character morality segment (5,000 ms), the affective state adjective (1,500 ms), and the affect reason (2,500 ms). These EMG segments were then divided into consecutive 100-ms bins to balance good temporal resolution and sufficient random error reduction (Van Boxtel, 2010). Average facial EMG activity during these bins was reported as percentage of the average pre-stimulus baseline activation, because this reduces random variance both within and between subjects (Van Boxtel, 2010). (Appendix for Fig. A2 shows continuous average activation for the four conditions in 100 ms bins for an entire trial).

Following our preregistration, we analyzed the three critical segments (character morality, affective state adjective, and affect reason) with separate Linear Mixed Models using R (version 4.3.1, R Core Team, 2023) and packages lme4 (version 1.1–34, Bates et al., 2015) and lmerTest packages (version 3.1–3, Kuznetsova et al., 2017). Instead of using custom *t*-tests we used the emmeans package (version 1.8.7, Lenth, 2023) to obtain the average corrugator activity during the critical segments and perform pairwise comparisons. The item- and participant variance were estimated simultaneously, resulting in a cross-classified model (Quené & van den Bergh, 2004; 2008). For each segment, we constructed models for the corrugator data by iteratively adding relevant components and testing for significant model improvement at each addition (using the likelihood ratio (−2LL difference chi-square) test, *p* < .05). We only kept components whose addition explained a significant amount of variance or were necessary to test hypothesized interactions (Winter, 2020). Components that did not significantly improve the model were dropped in the next iteration (H. van den Bergh, personal communication and grounded in Seasholtz & Kowalski, 1993; Struiksma et al., 2022).

Because we were interested in average corrugator activity and its development over time, we used a growth curve model approach (Mirman, 2014; Peck & Devore, 2011) to capture linear, quadratic, and cubic trends in our EMG signal. We first modeled participants and items as random factors and subsequently added character morality (moral vs. immoral) as a fixed factor in the model for the character manipulation segment, and character morality (moral vs. immoral), valence (positive vs. negative), and the 2-way interaction as fixed factors in the model for the affective state adjective and affect reason segments. Afterwards the interaction was added to the random part of the model as a random slope. Next, linear, quadratic, and cubic trends were added as covariates in the fixed part of the model. Time trend (e.g., linear) components were added per condition to maintain flexibility in building the model, and to avoid forcing the model to fit, for example, a linear trend for all conditions when only one condition contained a significant linear component. All trend components were centered to avoid correlation between trends (fixed effects final model intercepts therefore reflect the average corrugator activity across the entire segment, not the level at which corrugator activity intercepts the y-axis). By using trends up to the cubic component, we achieved some flexibility to fit responses without over-fitting or losing explanatory power (Mirman, 2014). Because we were particularly interested in temporal developments, we added random slopes for subjects for each time trend that initially improved the model (as well as standard random intercepts for subject and item). While fixed and trend components were fitted with a resolution of 100 ms, the associated parameter estimates (e.g., *b* for a linear slope) are reported on a 1-s basis. The significant interactions were post-hoc analyzed using pairwise comparisons with Sidak adjusted *p*-values. A summary of all model comparisons can be found in our online OSF repository and the final models are reported below.

#### Exploratory analyses

In our preregistration we suggested two exploratory analyses. The first suggestion was to analyze the correlation between the EMG signal and the embedded morality judgment rating task. Rather than analyzing this correlation for all three EMG segments, we focused on the character morality segment as this segment has the closest link to the ratings. The second suggestion was to correlate the fairness rating to the EMG activity. However, we decided to analyze only the behavioral data of the fairness rating, given the unexpected EMG results. We also included a custom made Justice Trait questionnaire as an exploratory task to measure individual differences on several of our outcome measures.

##### Analysis of the embedded morality judgment rating task

Using two Python scripts, we processed the Presentation logfile of each participant and extracted the embedded morality judgment ratings as well as the response to the comprehension questions. For each stimulus and immediately after the morality segment, describing our main character’s moral or immoral behavior, the embedded morality judgment rating task requested participants to rate the behavior of the main character on a 7-point scale (1 = “very bad” to 7 = “very good”). We were curious to see to which extent these behavioral ratings would correlate with participants’ frowning behavior at the time they were reading the morality segment. We focused on the peak of the differential effect in the morality segment, which occurred 3 to 4 s after the onset of the segment. We used Pearson’s product-moment correlation coefficient and looked at each participant’s average corrugator response during the peak of the morality segment and their behavior rating response immediately after this same segment. For the correlation analysis, we took the logarithm of the corrugator signal, because the results were not normally distributed. There were some trials where the participants did not give a response and some trials that were excluded as part of the baseline procedure described above. This resulted in a total data loss of 3.6%.

##### Analysis of justice trait questionnaire

A reliability analysis indicated that statements 2, 12, and 13 of the questionnaire were negatively correlated to the other items due to reverse phrasing (i.e., “I can feel sorry for people who behave badly”; see our online OSF repository for a full list of statements). After reverse coding these three statements, the total of all 17 statements measured the construct of “sensitivity to justice and fairness” (Cronbach’s α = .79). We then centered the scores (ranging from −2 to 2) so that a positive score corresponded to participants valuing the principle of “wanting positive things for good people and negative things for bad people.” A higher rating implied assigning more value to justice. Each participant’s average rating on these questions was then calculated to function as their personal justice trait score, as if their sensitivity were a personality trait.^2^ One outlier was removed using the Median Absolute Deviation method for outlier detection. As an exploratory analysis, we added the justice trait score as a factor to the final models of the character morality, state adjective, and affect reason analyses, testing for significant model improvement at each addition using the likelihood ratio as in our main analyses.

##### Analysis of story fairness

Rating responses in our Story Fairness Questionnaire corresponded to values from 1 (“very unfair”) to 7 (“very fair”) with the median being regarded as a neutral rating. Thus, the higher the score, the fairer the narrative’s ending was judged. To facilitate interpretation, we centered the ratings yielding −3 (“very unfair”) to 3 (“very fair”) and 0 being neutral. A combined amount of 87 ratings from seven different participants were excluded from the analysis due to an error in the creation of the four versions of the Story Fairness Questionnaire (corresponding to the four versions of stimuli lists). These issues were quickly fixed so the data of all other participants was complete. Furthermore, there was one participant whose ratings on all 64 narratives were excluded from analysis as everything was rated as neutral. This resulted in a total data loss of 4.1%. The Story Fairness rating responses allowed us to explore if and how the hypothesized effect of fairness-based evaluation would be reflected in a self-report measure as evaluation tends to occur on a more conscious level as well. Therefore, we built a linear mixed model focused on the interaction between the fixed factors morality condition (moral, immoral) and critical event valence (positive, negative) and with subject and item as random effects, with the Story Fairness ratings as the outcome measure. We performed pairwise comparisons, Sidak adjusted, to inspect significant effects between the conditions. As an exploratory analysis we added the justice trait score as a factor to the model of the story fairness ratings, testing for significant model improvement using the likelihood ratio as in our main analyses.

## Results

### Character morality

**Fig. 2:**
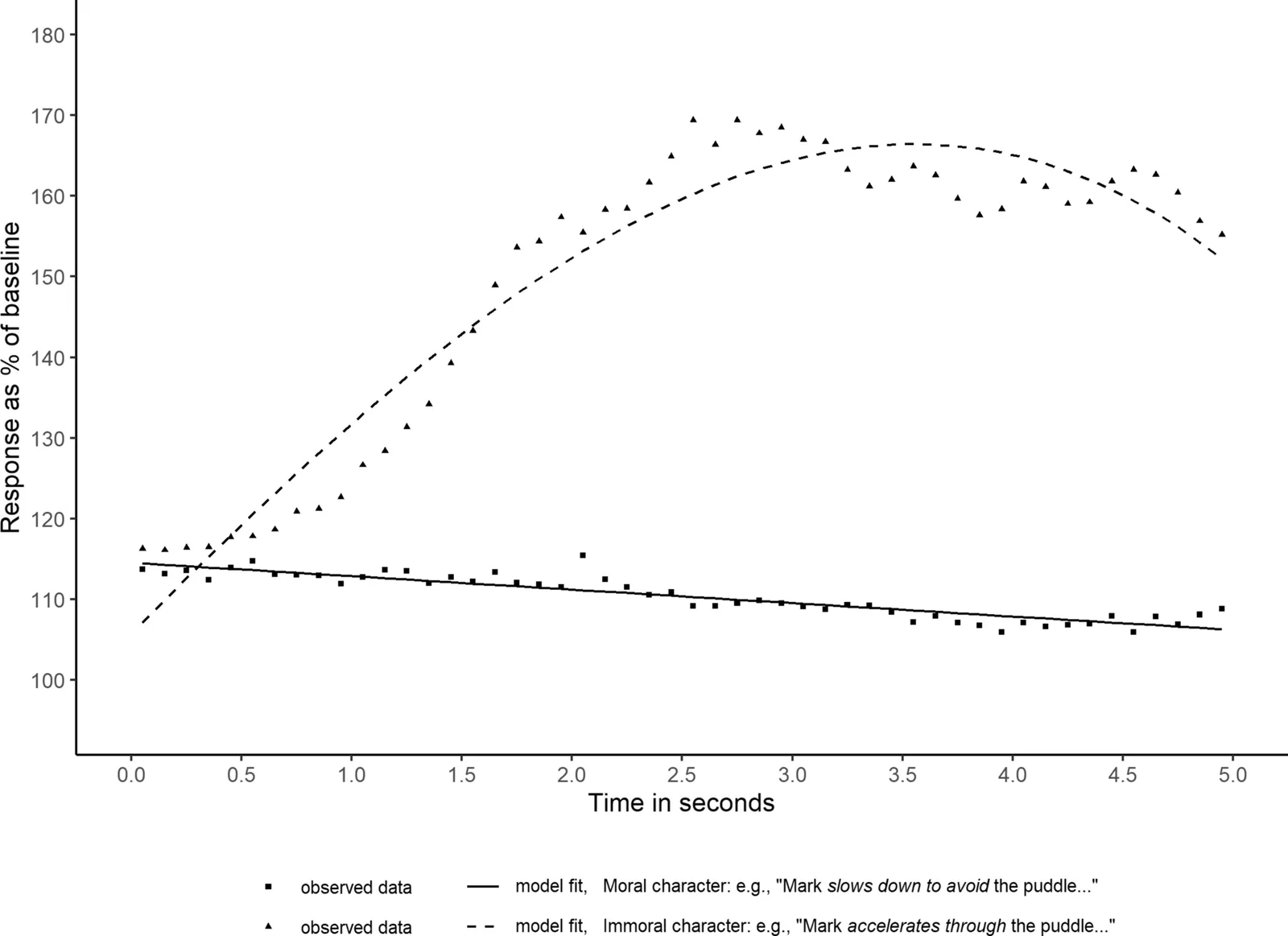
Observed averages of corrugator responses to character morality. Average corrugator response at the character morality segment in response to the presentation of moral and immoral characters, plotted by time. The growth curve model prediction is overlaid

As predicted, reading descriptions of immoral behavior elicited substantially more frowning than moral behavior.^3^ Figure 2 shows a clear differential effect of character morality (e.g., moral vs. immoral) on average corrugator activity. The statistical analysis corroborated this with a significant main effect of character morality (*b*_immoral-moral_ = 49.51, *z* = 4.15, *p* < .001, 95% CI [26.11, 72.91]). In this time window descriptions of immoral behavior also elicited a significant linear increase in corrugator activity, i.e., increased frowning (*b* = 12.25, *z* = 15.25, *p* < .001, 95% CI [10.68, 13.83]), as well as significant quadratic and cubic components (Table A1). Descriptions of moral behavior elicited a significant linear decrease in corrugator activity, indicating a subtle relaxation (*b* =  −1.66, *z* =  −5.18, *p* < .001, 95% CI [−2.29, −1.03]). This demonstrates that the character morality manipulation in our narratives was successful, replicating findings of the two previous studies where identical character morality segments were used ('t Hart et al., 2018, 2019).

### Affective state adjective

**Fig. 3:**
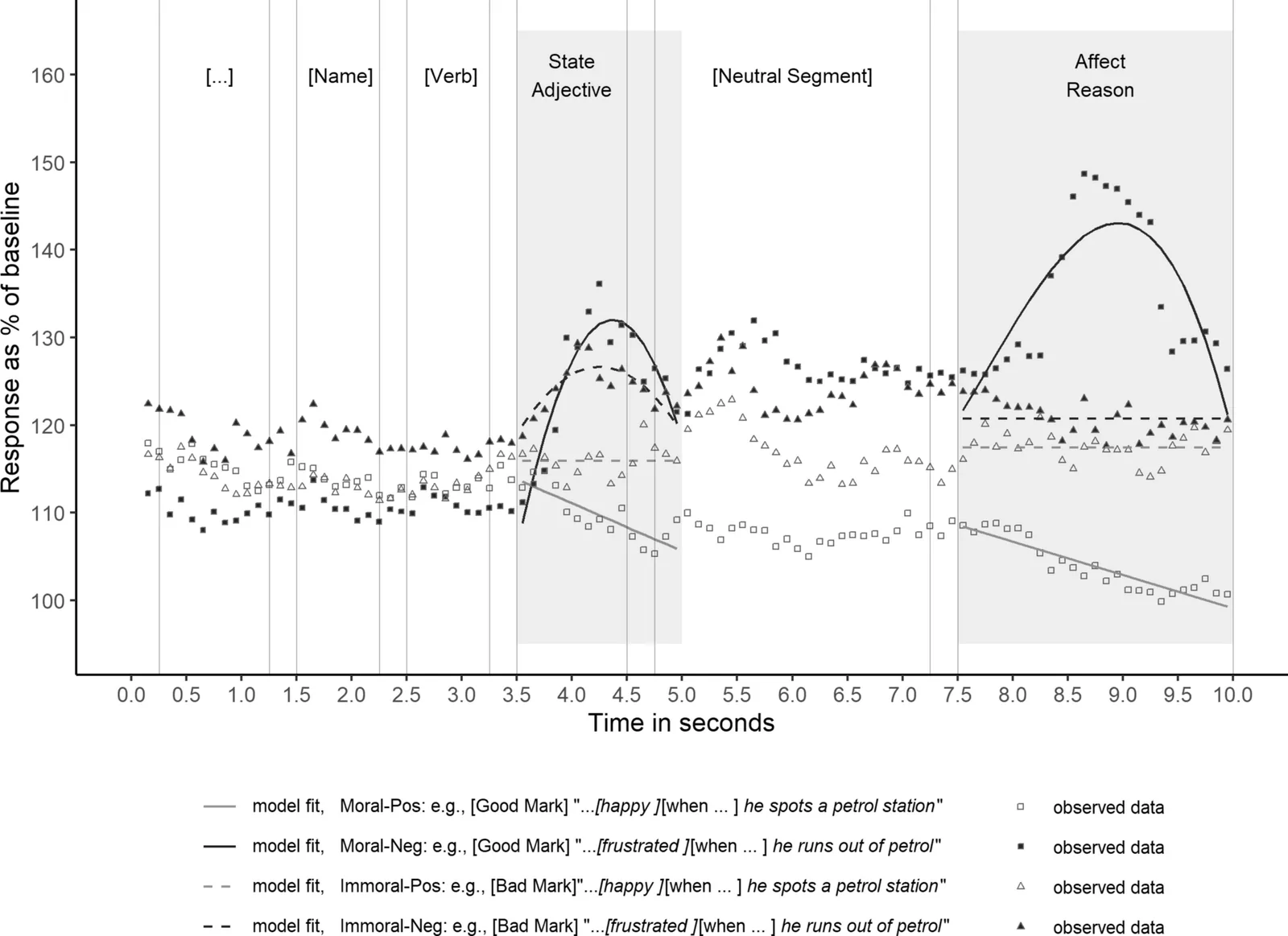
Observed averages of corrugator responses to the affective event. Average corrugator response at the two critical segments: affective state adjective and affect reason (highlighted), for moral and immoral characters ascribed with positive and negative adjectives, plotted by time. The vertical lines indicate the onset and offset of other segments, including 250 ms intersegmental intervals. The growth curve model prediction is overlaid

The affective state adjective segment refers to the main character’s affective state (e.g., happy vs. frustrated) as a result of something that is explained later in the reason segment. The affective state adjective discloses the valence of the affective event that befalls to that character. Figure 3 shows the average corrugator activity during a 10-s window, with the two highlighted pre-selected segments that were used for our analyses. The first highlighted segment refers to the window used to statistically analyze the response elicited by the affective state adjective and shows the fitted regression lines. The statistical analysis of the average corrugator activity in this 1.5-s window showed a significant main effect of character morality, as well as event valence (Table A2). Relevant to our predictions, also a significant interaction emerged between character morality and valence (*b* =  −11.36, *z* =  −5.83, *p* < .001, 95% CI [−15.18, −7.54]). There were also some significant time trends. Because our predictions were related to the corrugator activity in response to positive and negative events, we will discuss the moral and immoral conditions separately below.

#### Moral characters

The first shaded segment of Fig. 3 shows a clear differential pattern for the moral characters (square markers); the solid lines indicate the fitted growth curve model. We found that average corrugator activity was higher when moral characters were ascribed with negative adjectives, i.e., a description of a good person feeling frustrated, than with positive adjectives, i.e., a description of a good person feeling happy (Table 2; *b*_*neg-pos*_ = 22.68, *t*(55,86) = 16.44, *p*_*Sidak*_ < .001, 95% CI [19.24, 26.11]). For negative adjectives, the model also contained a linear component, which improved the model, but did not prove to be significant (Table A2). However, its negative cubic component was significant and indicates a rise-fall pattern (Table A2). For positive adjectives, the significant negative linear component indicates relaxation of the corrugator. Together these average activity and time trends indicate a phasic increase in frowning when reading about a good person feeling frustrated and a decrease in frowning when reading about a good person feeling happy. These results about moral characters confirm our predictions, replicate previous findings, and are in line with the postulated joint effect of simulation and evaluation on the corrugator.

**Table 2:** Estimated marginal means of the interaction between character morality and event valence for the state adjective and affect reason segment

	State adjective		Affect reason	
Character morality* Event valence	EMM	95% CI	EMM	95% CI
Moral negative	131	(120, 142)	142	(131.5, 152)
Moral positive	109	(97.4, 120)	103	(92.6, 113)
Immoral negative	126	(115.1, 138)	120	(110, 131)
Immoral positive	115	(103.9, 126)	116	(106.1, 127)

*EMM *estimated marginal mean, *CI* confidence interval

#### Immoral characters

For immoral characters, we predicted a net *relaxation* of corrugator activity at negative state adjectives, i.e., a bad person feeling frustrated, or at least to find the corrugator activity to *fall below* that at positive state adjectives, i.e., a bad person feeling happy. Surprisingly, as shown in Fig. 3 (triangles), we observed the opposite pattern. Average corrugator activity in response to positive and negative adjectives describing immoral characters showed a similar, although attenuated, pattern, as we found for moral characters (Table 2; *b*_*neg-pos*_ = 11.31, *t*(55,86) = 8.21, *p*_*Sidak*_ < .001, 95% CI [7.88, 14.75]). For negative adjectives, the quadratic time component improved the model fit but did not prove to be significant.

### Affect reason

The event reason segment is the final segment of the narrative and describes the circumstances that led to the main character’s affective state. In two prior studies we found that reading the affect reason appeared to elicit renewed phasic corrugator activity,^4^ similar to the differential corrugator response at the adjective but of a larger magnitude ('t Hart et al., 2019, 2021). In line with our previous work, we examined whether our expected corrugator pattern with boosted evaluation was visible for this segment. The final model included significant main effects of character morality and event valence in addition to a significant interaction between the two (Table A2). Because our expectations concerned the corrugator response related to the event valence but depending on the character morality, we will again discuss the pairwise comparisons of the interaction and the time trends for the moral and immoral conditions separately.

#### Moral characters

For moral characters experiencing positive and negative events, we see a renewed differential pattern emerging (squares in Fig. 3). Pairwise comparisons revealed that average corrugator activity was much higher for negative affect reasons than for positive affect reasons (Table 2; *b*_*neg-pos*_ = 38.99, *t*(93,27) = 31.24, *p*_*Sidak*_ < .001, CI [35.88, 42.10]). For the negative affect reason, the final model also included non-significant linear and cubic components fitting the increase in frowning over time. At the positive affect reason, however, we found a significant linear decrease, indicating relaxation (Table A2). This implies that we indeed see the same valence-based differential pattern that we found for the state adjectives describing moral characters but now at a slightly larger scale.

#### Immoral characters

When immoral characters experience positive and negative events we do not observe a change in corrugator response (triangles in Fig. 3) when the event reason is revealed, both conditions are fitted by a horizontal line. Pairwise comparisons indicated that the average corrugator activity did significantly differ between the two events, albeit with a small difference (Table 2; *b*_*neg-pos*_ = 3.85, *t*(93,27) = 3.94, *p*_*Sidak*_ = .003, CI [1.41, 6.29]). Instead of a renewed differential response, the difference between positive and negative events seemed smaller at the event reason compared to the state adjective. The corrugator activity for immoral characters experiencing negative events, i.e., a bad person feeling frustrated, did *not fall below* that for experiencing positive events, i.e., a bad person feeling happy. Similar to the corrugator patterns at the adjective, this is again the opposite of the expected pattern.

### Exploratory results

#### Morality judgment rating task

For exploratory reasons, we also look at the correlation between the EMG signal at the character morality segment and the ratings on the embedded morality judgment task, a self-report measure of the readers’ conscious evaluation of a character’s behavior that occurred immediately after the character morality segment. By asking our readers to indicate how good or bad the behavior of our characters was, we aimed to emphasize their moral status and therefore enhance fairness-based moral evaluation when they read about the characters’ affective states afterwards. We found a weak negative correlation between the morality judgment ratings and the logarithm of average corrugator activation during the 1-s peak of the morality segment (*r*(3,699) =  −.23, *p* < .001). This indicates that lower ratings, reflecting stronger self-reported disapproval, were associated with higher average corrugator activity, i.e., more frowning. This is what we would logically expect. Although frowning responses are presumed to indicate unconscious and implicit moral judgement and the ratings are an indication of conscious judgement, this loosely supports the notion that participants frowning responses are indeed indicative of how they judge characters’ behavior in terms of valence.

#### Justice trait questionnaire

**Fig. 4:**
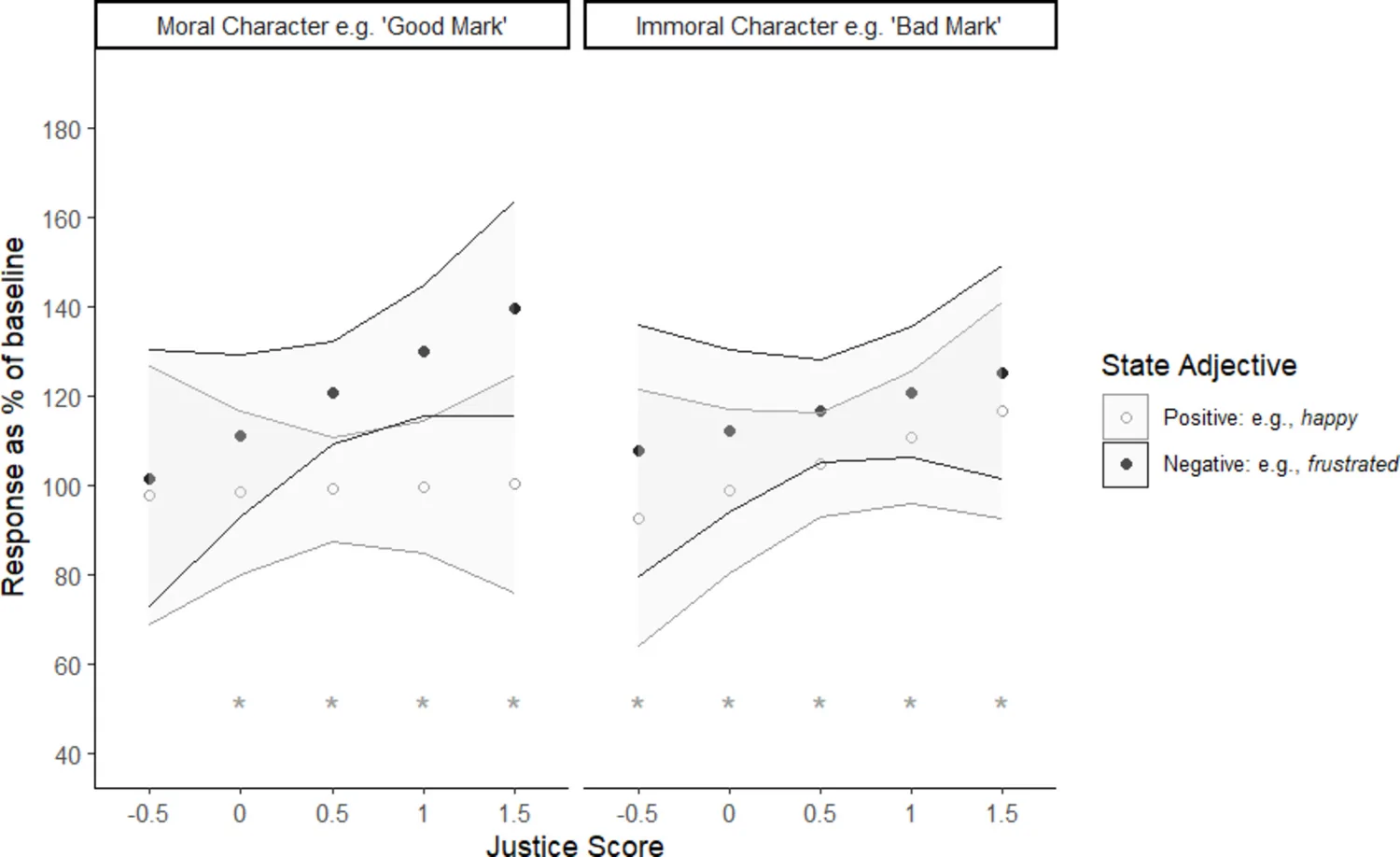
Observed averages of corrugator responses in the state adjective segment for different justice trait scores. Average corrugator responses to the positive and negative state adjectives in the state adjective segment, for different justice trait scores and split for moral and immoral characters. Error bands represent 95% confidence intervals and * indicates significant pairwise comparisons (*p* < .05)

We also added the justice trait score to the final corrugator activity models. Adding the justice score to the final character morality model did not improve the fit of the model (*p* = .07, see our online OSF repository for a full report of the analyses). We then added the justice trait score to the final model of the state adjective, which did improve the model fit (*p* < .001, see Table A4 for details on the full model). The subsequent three-way interaction between character morality, event valence and justice trait score was significant (Fig. 4; *b* =  −20.93, *z* =  −4.97, *p* < .001, CI [−29.18, −12.68]). Because the justice trait score is a continuous variable, we probed this interaction at specific values for the justice score with increments of 0.5, as participants’ scores ranged from −0.5 to 1.5. As shown in Fig. 4, comparing people with high and low justice trait scores yielded different responses in the moral and immoral conditions.

In the moral condition, participants with a negative justice trait score (i.e., people who do not agree with wanting positive things for good people and negative things for bad people) do not show a significant differential response toward the positive and negative state adjectives (*p*_*Sidak*_ = .24 at a justice score of −0.5, see Table A5 for all the details on the pairwise comparisons). Participants who were indifferent or agreed with wanting positive things for good people and negative things for bad people, i.e., those with justice score of zero or higher, frowned more in response to negative state adjectives compared with positive state adjectives (*p’s*_*Sidak*_ < .001 at a justice scores 0 to 1.5; Table A5). Particularly, the higher participants valued justice, the more they frowned at negative state adjectives (*slope*_*MoralNeg*_ = 18.96, *slope*_*MoralPos*_ = 1.31, *Δslope*_*Moral*_ = 17.66, *t*(54,987.80) = 6.65, *p*_*Sidak*_ < .001, CI [10.67, 24.65]). As per our multiple-drivers model, frowning at negative state adjectives for moral characters could be the net result of negatively valenced simulation (i.e., simulating the negative word or situation) and additional negative evaluation (i.e., finding something unfair). Our justice trait score seems to influence the degree of frowning in this case, hinting at a possible modulation of negative evaluation. Only participants who value a “sense of justice” frown at unfair events that a moral character experiences.

Interestingly, in the immoral conditions, all participants, irrespective of their justice score, frowned more in response to negative state adjectives than in response to positive state adjectives (*p’s*_*Sidak*_ < .008; Table A5). This pattern can be explained by negative simulation rather than evaluation. Recall that evaluation was expected to counteract the simulation force, yielding no differential responses or even a flipped waveform pattern, which is clearly not the case. Moreover, the higher the justice score, the higher the overall corrugator activity (*b* = 10.66, *z* = 4.02, *p* < .001, 95% CI [5.46, 15.85]).

**Fig. 5:**
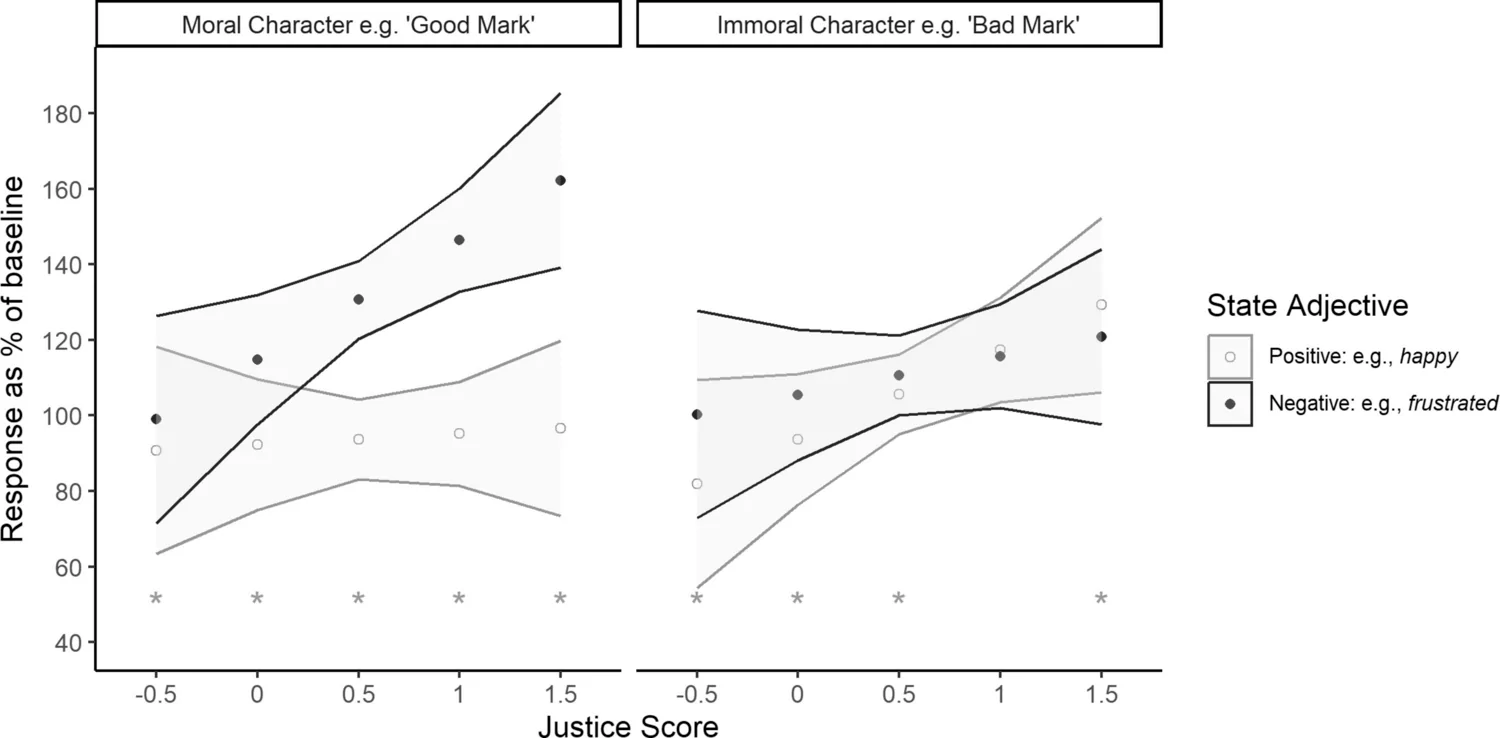
Observed averages of corrugator responses in the affect reason segment for different justice trait scores. Average corrugator responses to the positive and negative events in the affect reason segment, for different justice trait scores and split for moral and immoral characters. Error bands represent 95% confidence intervals and * indicates significant pairwise comparisons (*p* < .05)

As a final step, we also added the justice trait score to the final model of the affect reason, which did not converge. However, when we removed the random intercept for the cubic component for negative affect in moral characters, and included the justice trait score, this did improve the model (*p* < .001; see Table A6 for details on the full model). The three-way interaction between character morality, event valence, and justice trait score was also significant in this model (Fig. 5; *b* =  *−*42.13, *z* =  −12.93,* p* < .001, CI [−48.80, −35.47]). We then probed the three-way interaction, using the same values of the justice trait score as in the previous exploratory analysis (−0.5 to 1.5). Again, people with high vs. low justice trait scores showed different response patterns in the moral and immoral conditions.

In the moral conditions, similar to the responses at the state adjective, our justice trait score seems to influence the degree of frowning. All participants frowned more in response to negative events compared with positive events (*p’s*_*Sidak*_ < .005 at a justice score from −0.5 to 1; Table A7). Moreover, this difference was more substantial the more strongly the participants valued a “sense of justice” (*slope*_*MoralNeg*_ = 31.63, *slope*_*MoralPos*_ = 2.91, *Δslope*_*MoralNeg-Pos*_ = 28.72, *t*(91,837.37) = 11.96, *p*_*Sidak*_ < .001, CI [22.40, 35.03]).

Interestingly, in the immoral conditions, participants with a low-to-moderate justice score frowned significantly more in response to negative events than to positive events that were provided as the reason for characters’ affective states (*p’s*_*Sidak*_ < .001 at justice score from −0.5 to 0.5; Table A7). This pattern can again be explained by negative simulation rather than evaluation. However, note that for participants with a higher justice score the pattern starts to flip, as indicated by a significant decreasing difference in slopes for negative and positive events (*slope*_*ImmoralNeg*_ = 10.25, *slope*_*ImmoralPos*_ = 23.66, *Δslope*_*ImmoralNeg-Pos*_ =  −13.42, *t*(91,831.81) =  −5.59, *p*_*Sidak*_ < .001, 95% CI [−19.73, −7.10]). For participants with the highest justice score we see that they frowned significantly more in response to positive events compared to negative events (*p*_*Sidak*_ = .22 at a justice score of 1 and *p*_*Sidak*_ < .001 at a justice score of 1.5; Table A7). This is the pattern that we had hypothesized to find overall and would align with counteracting forces postulated under the multiple-drivers account: positive evaluation (“fair”) but negative simulation for negative events befalling an immoral character.

#### Story fairness questionnaire

The Story Fairness Questionnaire was administered after the EMG part of the experiment to gain more insight into conscious evaluation of the fairness of our narratives and asked our participants to re-read each narrative stimulus and then rate the fairness of each. We investigated whether the hypothesized effect of fairness-based evaluation would be reflected in this self-report measure. To do so, we built a linear mixed model, again focusing on the interaction between the fixed factors character morality and critical event valence, but now with the story fairness ratings as the outcome measure. Our mixed model analysis (Table A8) showed that the interaction between character morality and event valence was significant (*b* = 6.44, *z* = 80.85, *p* < .001, 95% CI [6.29, 6.60]). Pairwise comparisons revealed that ratings were lower, i.e., indicating more ‘unfair’ judgements, for negative than positive events in the case of moral characters (*b*_*neg-pos*_ =  −3.50, *t*(3,568.25) =  −62.11, *p*_*Sidak*_ < .001, CI [−3.64, −3.36]), whereas the pattern was reversed for the immoral ones (*b*_*neg-pos*_ = 2.94, *t*(3,566.56) = 52.24, *p*_*Sidak*_ < .001, 95% CI [2.80, 3.08).

Interestingly, the average ratings precisely show the evaluation-based valence patterns that we were expecting to find in the EMG signals: for moral characters we see positive/fair ratings for “good Mark happy” and negative/unfair for “good Mark frustrated,” but the opposite for immoral characters.

**Fig. 6:**
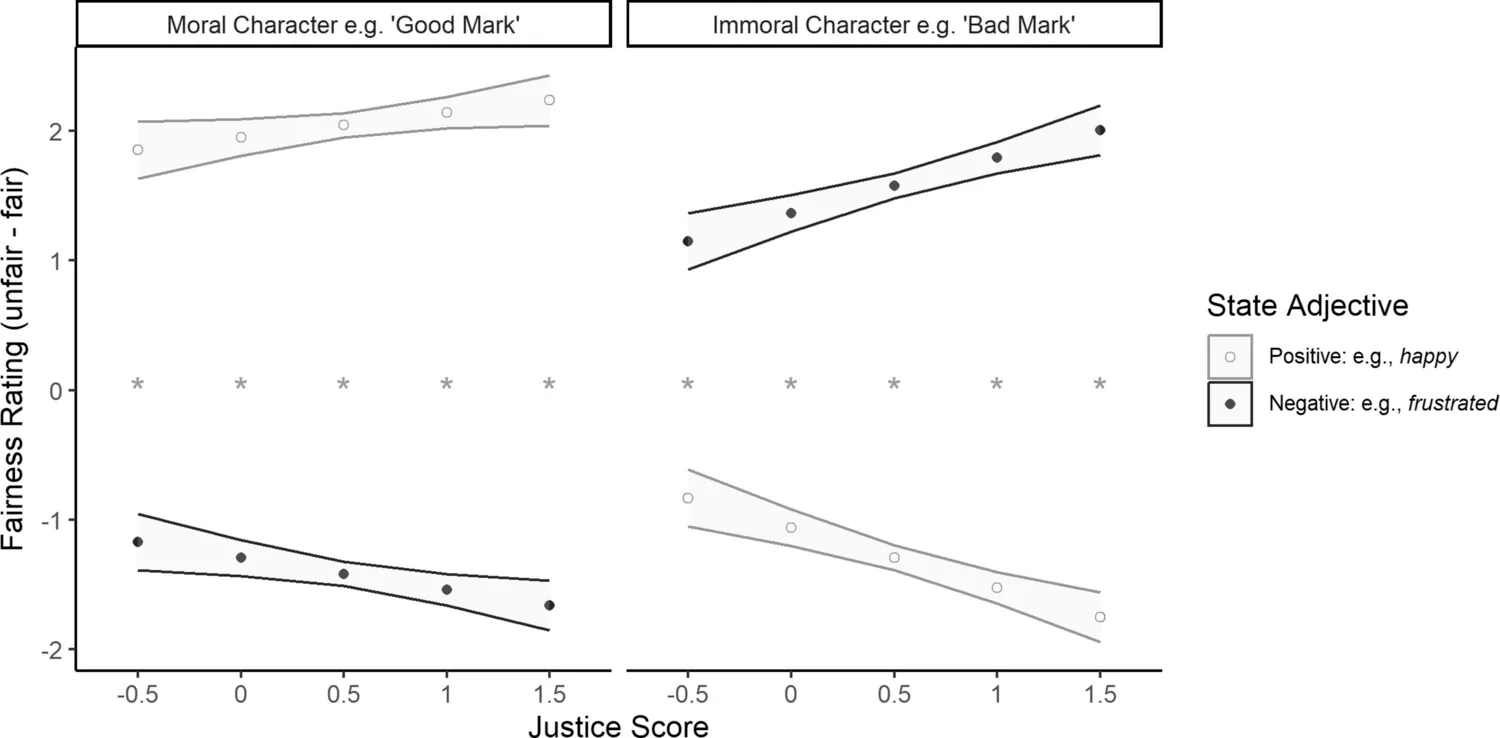
Observed averages of story fairness ratings for different justice trait scores. Average Story Fairness ratings to the positive and negative events, for different justice trait scores and split for moral and immoral characters. Error bands represent 95% Confidence Intervals and * indicates significant pairwise comparisons (*p* < .05)

We then added the justice scores to the model and this significantly improved the model fit (*p* < .001, see Table A8 for details on the full model). The three-way interaction between character morality, event valence and justice trait score was significant in this model (Fig. 6; *b* = 1.33, *z* = 7.97,* p* < .001, 95% CI [1.00, 1.65]). We then probed the three-way interaction, using the same values of the justice trait score as in the previous exploratory analysis (−0.5 to 1.5). People’s justice trait scores seemed to moderate the degree of fairness ratings that the narratives received.

For all participants, irrespective of their justice score the difference between ratings for the negative and positive events was significant (*p’s*_*Sidak*_ < .001, see Table A9). What seems to happen is that the difference in rating between negative and positive affect reasons increases as the justice score increases, as indicated by significant differences in slopes for negative and positive events for moral characters (*slope*_*MoralNeg*_ =  −0.24, *slope*_*MoralPos*_ = 0.19, *Δslope*_*MoralNeg-Pos*_ =  −0.44, *t*(3,622.90) =  −3.72, *p*_*Sidak*_ = .001, CI [−0.74, −0.13]), as well as immoral characters (*slope*_*ImmoralNeg*_ = 0.43, *slope*_*ImmoralPos*_ =  −0.46, *Δslope*_*ImmoralNeg-Pos*_ = 0.89, *t*(3,622.53) = 7.60, *p*_*Sidak*_ < .001, 95% CI [0.58, 1.20]). These results suggest that participants that indicated they strongly valued a “sense of justice” gave more extreme ratings, i.e., stronger fair judgments in fair situations and stronger unfair judgments in unfair situation.

## Discussion

Our emotion systems become active when we process affective language, often resulting in measurable facial muscle responses. Yet, the cognitive mechanisms underlying this activation remain a subject of debate. Across three prior studies ('t Hart et al., 2018, 2019, 2021), we investigated how mental simulation and moral evaluation might influence the corrugator supercilii (i.e., the “frowning muscle”) and found converging evidence supporting the multiple-drivers account of affective language comprehension. This account posits that simulation and evaluation operate as joint forces for moral characters but as counteracting forces for immoral ones. Although previous results were most consistent with this account, they could not conclusively demonstrate that simulation and evaluation function as separable influences, nor could they verify the predicted interplay between them. The present study was designed to address these gaps by selectively amplifying the evaluation component while keeping all other factors constant. Using the same narratives as in our 2019 study ('t Hart et al., 2019), we added a morality judgment rating task embedded within our stimuli intended to encourage readers to actively evaluate the characters’ moral status. As in prior work, the primary focus was on the corrugator response to affective state adjectives. Crucially, we predicted a divergence in response patterns for immoral characters: somewhat more frowning when they experienced positive emotions than negative ones—the opposite of the expected pattern for moral characters. Such a finding would provide stronger evidence that simulation and evaluation can be experimentally dissociated and may modulate facial muscle responses as separate forces, consistent with the multiple-drivers account.

### Reviewing the multiple-drivers account

As in our previous work ('t Hart et al., 2018, 2019), the morality segments again elicited the expected corrugator responses: increased frowning when reading about immoral behavior and relaxation when reading about moral behavior. These findings reaffirm the validity of our morality manipulation and establish a consistent baseline for interpreting subsequent affective responses.

When reading about characters’ affective states as expressed through adjectives, such as “happy” or “frustrated,” we found that readers frowned more when moral characters were described with negative adjectives than with positive ones. This pattern replicates earlier findings ('t Hart et al., 2019) and aligns with facial EMG research on emotional word processing (Fino et al., 2016; Foroni & Semin, 2009; Larsen et al., 2003). The results support the idea that simulation and evaluation can converge: negative adjectives evoke both negative emotion simulation and negative evaluation (sense of unfairness) when applied to moral characters, amplifying the frowning response. Conversely, positive adjectives in this context elicit both positive simulation and positive evaluation (sense of fairness), leading to relaxation.

In contrast, for immoral characters, we had hypothesized that fairness-based evaluation would override emotion simulation, leading to greater frowning for positive adjectives (unfair) and reduced frowning for negative adjectives (fair). Nevertheless, this expected flipped pattern did not emerge in our main analyses. Instead, the data revealed more frowning in response to negative adjectives than positive ones, mirroring the pattern seen in moral conditions and contradicting the predicted dominance of evaluative processing. This raises important questions about the relative influence of simulation and evaluation within the multiple-drivers account.

Importantly, these findings cannot be explained by models assuming a single underlying process. If simulation alone were driving corrugator responses, we would expect adjective valence-consistent frowning regardless of character morality. If evaluation alone were responsible, moral status would fully dictate the corrugator response. Instead, we observed that both character type and adjective valence influenced outcomes, supporting a multiple-drivers account although one that may require refinement.

Further complexity arose in the affect reason segment, which provided the reason for each character’s emotional state. As in previous studies ('t Hart et al., 2019, 2021), we observed renewed corrugator modulation in the moral condition in response to valence: more frowning for negative events, less for positive ones. In the immoral condition, rather than renewed divergence as in our 2019 study, corrugator patterns appeared to largely continue the differential trend we found at the adjective. Unlike our expectations of a flipped pattern, we observed attenuated patterns, similar to those of the moral condition. This suggests that corrugator activity related to emotional evaluation is not static, but rather, it dynamically unfolds as additional contextual information becomes available. Nevertheless, due to the distributed nature of affectively-salient information in this segment, precise time-locking of emotional effects is less reliable, and findings should be interpreted with caution.

Overall, the results reaffirm the multiple-drivers account, while also highlighting its limitations. Although it continues to offer a better fit than single-driver explanations, our findings suggest that simulation and evaluation interact in more complex ways than initially theorized. In particular, the absence of the predicted boosted evaluation in the immoral condition challenges the assumption that fairness-based evaluation always increases linearly in response to task demands or narrative cues.

**Fig. 7:**
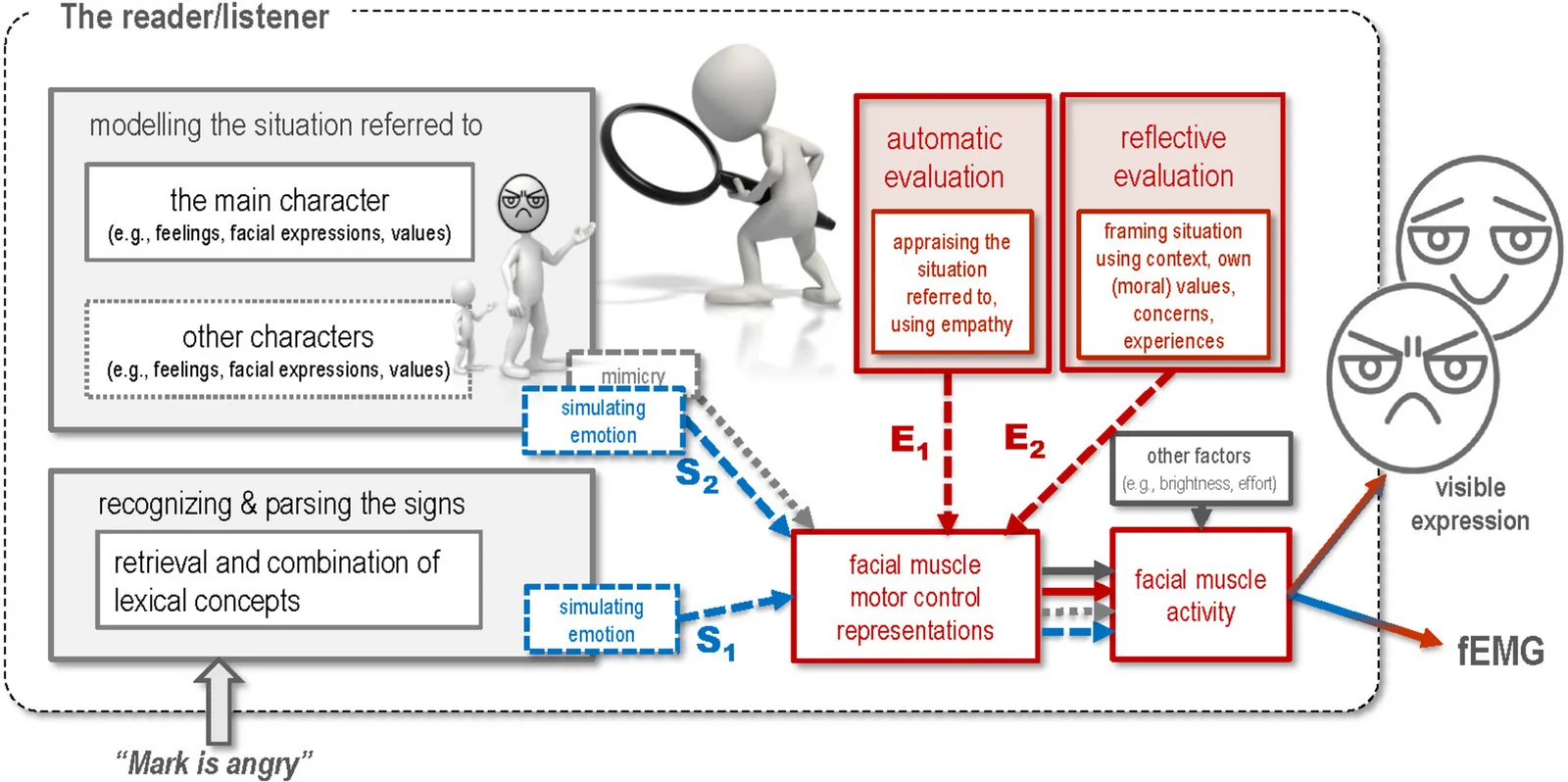
Hypothetical updated multiple-drivers account. The hypothetical updated multiple-drivers account, adapted from the broader fACL model (see Van Berkum et al., 2023), shows how facial muscle activity may be affected by both simulation and evaluation of affective language during processing. Additionally, the model recognizes that these drivers could be multifaceted and that there may be other factors, including mimicry, that drive our facial expressions

We therefore propose that moral evaluation is not uniformly fairness-based. One possibility is that readers may experience empathy even toward immoral characters, blunting the expected frowning response to unjustly positive outcomes, after all a person who behaves immorally is still a person. In real-life social cognition, people often hold mixed or ambivalent feelings (Willems, 2023), and such multifaceted responses may surface even in controlled experimental settings. Evaluation from a fellow-human perspective might be responsible for an additional facet of evaluation based on empathy, i.e., positive emotions, such as “happy,” cause corrugator relaxation whereas negative emotions, such as “frustrated,” cause increased frowning regardless of one’s moral status. With our stimuli, for instance, true fairness-based evaluation might occur only after the final piece of information is revealed by the affect reason segment. Within the multiple-drivers account, this suggests a need to accommodate multifaceted evaluations, where both empathy- and fairness-based evaluations co-exist, interact, or compete, depending on context and timing. Another possibility may be that the corrugator initially reflects mainly situation model simulation that is only slightly modulated by rapid and automatic evaluation, such that the net corrugator response mostly aligns with the valence of the adjective, only counteracted to some extent in the immoral conditions. The subsequent continuation and eventual attenuation of the differential trend at the neutral and reason segment could reflect the unfolding of a secondary more reflective evaluation process (Cunningham & Zelazo, 2007); Moors et al., 2013), as readers start to reason about the content in the larger context and based on their own moral values: “Hey, I actually don’t mind that [Bad Mark] ends up frustrated,” and “ I don’t support that [Bad Mark] ends up happy”). As such, the multiple-drivers account could consist of a simulation driver and a multifaceted evaluation driver that ranges from automatic to reflective processing (Cunningham & Zelazo,^2007^). We cautiously demonstrate a hypothetical version of such an updated model in Fig. 7, where the evaluation driver is split into a rapid and automatic appraisal evaluation and a subsequent more conscious and reflective evaluation, although more empirical research should verify this model.

### The impact of the embedded morality judgment rating task

A key methodological deviation from our previous studies was the inclusion of an embedded morality judgment task, intended to enhance fairness-based evaluation during the processing of affective state adjectives. Contrary to our hypothesis, the resulting corrugator patterns suggested a reduction, rather than an amplification, of evaluative processing, particularly in the immoral condition. While the global timing of phasic EMG responses remained consistent, we speculate that the task may have inadvertently disrupted participants’ evaluative engagement by shifting attention away from the unfolding narrative and back toward an external judgment mode, hence dampening the contribution of the evaluation driver. In line with this idea, studies found discrepant ERP results in comparing the same moral and immoral materials in a comprehension task and a task that required explicit moral judgments, such that the explicit task reflected more dominant cognitive-semantic processes, whereas the implicit task reflected more affective evaluation processes (Kunkel et al., 2018; Leuthold et al., 2015).

Moreover, we note that readers could have perceived a disconnect in our narratives between characters’ earlier moral behavior and their affective states later on, which meant that our proposed fairness-based evaluation relied more on automatic evaluation or general moral intuitions (Haidt, 2001) than direct retributive logic, potentially weakening its influence. It is also plausible that actively reflecting on moral behavior during the rating task depleted the cognitive resources needed to fully integrate subsequent emotional information, a pattern reminiscent of attentional blink phenomena (Johns et al., 2008). This may have been particularly impactful in the immoral condition, where simulation-driven responses appear to have dominated early on, with evaluation effects emerging only later at the reason segment. The task may also have highlighted meta-awareness of our manipulations such that readers started evaluating more carefully: “Mark may have behaved badly, but it does not automatically mean that any subsequent negative affect is deserved. I should probably wait until I know the reason to determine whether the outcome is fair.” Thus, the task may instead have exposed the time-course of multifaceted evaluation such that automatic evaluation based on general moral intuitions regarding good or bad people may have contributed at the state adjective, while the effect of the morality manipulation, contributing to reflective evaluation, is postponed until the reason segment (Fig. 7). Taken together, these findings suggest that the embedded task, under the multiple-drivers account designed to boost evaluation, added cognitive and physical demands which may have unintentionally suppressed or altered evaluation. Whether through attentional load, narrative disengagement, or moral intuitions, our results suggest that the embedded rating task did not achieve its intended effect and may even have interfered the very process it aimed to enhance. This highlights the need for caution when integrating active judgment tasks within affectively sensitive narrative contexts.

### Exploring fairness and justice sensitivity

Interestingly, the self-reported fairness ratings of our narratives displayed the expected evaluation-based valence patterns, positive outcomes judged as fair for moral characters and unfair for immoral ones, which were also modulated by individual difference in justice sensitivity. The more strongly individuals valued a “sense of justice”, the more extreme fairness ratings they gave. Surprisingly, the corresponding corrugator responses did not always align with these explicit evaluations. This discrepancy highlights a crucial distinction between conscious, deliberate moral reasoning (Haidt, 2001) as measured by the ratings, and the more unconscious evaluative processes reflected in facial EMG (Barrett et al., 2007; Van Boxtel, 2010). Moreover, as discussed above, the discrepancy might also be due to the fact that the corrugator responses could reflect both automatic and reflective evaluation pertaining to different time frames of the response, while the rating is a single response. Although the Story Fairness Questionnaire confirmed that our narrative stimuli had the capacity to manipulate fairness-based evaluation in a moral context, these self-reports are susceptible to social desirability and task transparency effects, such as the meta-awareness of our manipulation mentioned above (Izard, 2009; Van Berkum, 2018), which might also partly explain the differences found between the outcome measures.

Notably, the results from our custom-made questionnaire measuring individual differences in justice sensitivity suggested that this trait further shaped corrugator activity. Participants with high justice trait scores, those who reported to strongly value fair outcomes showed greater frowning in response to negative affective states and events experienced by moral characters, consistent with additive effects of negative simulation and negative evaluation. In contrast, participants with lower justice scores showed no such modulation, suggesting a relative absence or attenuation of the evaluative force. In immoral conditions, all participants frowned more at negative states and the higher the justice score the more participants frowned, indicating that simulation might not be interacting with evaluation for this response as they were waiting for additional contextual information to become available. However, for participants highest in justice sensitivity, this pattern began to reverse at the affect reason segment, with greater frowning in response to positive outcomes, indicating a dynamic response and precisely the pattern we expected for fairness-based evaluation counteracting simulation. Though we acknowledge the Justice Trait Questionnaire’s exploratory nature as an unvalidated measure, our exploratory analyses show a clear pattern of systematic individual differences. Overall, our findings support a refined view of the multiple-drivers account, in which evaluation and simulation can be independently modulated and interact in dynamic, individual ways depending on narrative context and individual moral orientation.

## Conclusion

Together, our findings reaffirm the relevance of the multiple-drivers account for understanding affective responses to moral language, while simultaneously exposing its current limitations. Corrugator responses consistently reflected the emotional valence of characters’ described morally loaded behavior, confirming that narrative context can reliably engage simulation and evaluative processes. However, when these characters’ affective states and their reasons were introduced, predicting the net corrugator response proved far less straightforward, particularly in cases where simulation and evaluation were expected to exert opposing effects. This highlights a central challenge: the interplay between emotion simulation and moral evaluation is not only dynamic but also sensitive to context, timing, and individual differences.

Importantly, the current study illustrates that evaluation is likely multifaceted in nature. The impact of our embedded morality judgment rating task, intended to boost fairness-based evaluation, suggests that evaluative engagement can be unintentionally disrupted by task design, attentional demands, or narrative disjunctions. Moreover, our exploratory analyses of justice sensitivity revealed potentially meaningful individual variability: only participants with the strongest fairness orientations showed the predicted counteracting evaluation effects, particularly in response to immoral characters experiencing positive outcomes. This provides initial evidence that evaluative processing might not only be context-dependent, but also trait-modulated.

We argue that these findings point to the need for a more nuanced understanding of multifaceted evaluation within the multiple-drivers account. We propose that future studies of this account explicitly incorporate the possibility of competing or co-existing evaluative facets, such as fairness and empathy, or automatic and reflective processes, which may interact, override, or co-exist with simulation in shaping affective responses. Future studies may design new stimuli, perhaps in which moral behavior is more closely linked to the later outcome of the story, or with more precise time-locking of the event reason to EMG. Taken together, more targeted research will be needed to unravel how these facets interact over time, and how they are influenced by narrative structure, cognitive load, and individual moral orientation.

In conclusion, while our study further validates the multiple-drivers account as a valuable framework, it also underscores the complexity of moral emotion processing in language. A more comprehensive model that accommodates multifaceted evaluation and accounts for individual traits like justice sensitivity, such as the hypothetical updated model presented, will better reflect the richness of real-world affective experience. Continued exploration of these mechanisms holds promise not only for theories of affective language comprehension, but also for broader questions in moral cognition and social neuroscience.

## Supplementary Information

Below is the link to the electronic supplementary material.

Supplementary file1 (DOCX 716 KB)

## Data Availability

Our raw data files can be found at https://doi.org/10.24416/UU01-1PAHMB. All experimental materials can be found at https://doi.org/10.17605/OSF.IO/UENMR.

## Footnotes

1 Gender effects were not of theoretical interest for the current study and were therefore not included in the preregistration and analyses. Balancing the genders of our main characters was assumed to take care of potential gender-related biases in our data.

2 Because this task is a self-report measure, it should be noted that rating responses may reflect both participants’ personal values and beliefs concerning justice, as well as what they think is socially sensible or desirable.

3 The corrugator responses in our figures start slightly above the 100% baseline level. We suspect this reflects passive viewing of a neutral image during baseline as well as the effort demanded by reading the preceding introduction segment of the narratives.

4 In ‘t Hart et al. (2019), we performed a post-hoc analysis of the neutral segment in between the affective state adjective and affect reason segments. We also analyzed the current neutral segment (4.75–7.5-s window in Fig. A1) and found a stable pattern of activity. We observed a horizontal line in response to all conditions except for the immoral characters experiencing positive events, which showed a subtle decrease. All average corrugator responses differed significantly (*p’s* <.008). Details of the analysis of the neutral segment can be found in Table A3 and Fig. A1.

## Code availability

All code and analyses files can be found at https://doi.org/10.17605/OSF.IO/UENMR. The experiment and planned analyses were preregistered when data collection had just started as part of an RMa thesis project, but the data had not yet been quantified, constructed, observed, or reported by anyone. A copy of the preregistration can be found on our GitHub repository (https://github.com/mstruiksma/EMG4), which is also linked to our OSF repository.
