# Supplementary material for: Corrugator activity reflects integrated simulation and multifaceted evaluation when processing affective language: Frowning responses to reading about emotions of morally good and bad characters
Source: Cogn Affect Behav Neurosci. 2026 Aug 7;26(5):2118–38. doi: 10.3758/s13415-026-01464-8 (PMC13615145; doi:10.3758/s13415-026-01464-8)
Supplement: Supplementary file 1 [file 13415_2026_1464_MOESM1_ESM.docx]

# Appendix

Table A1

Character Morality Linear Mixed Models Analysis Summary.

|  | Character Morality Segment  (M12, *df* = 186989) | | | |
| --- | --- | --- | --- | --- |
| Predictors | Estimate | (95% CI) | *z* | *p* |
| (Intercept-Moral) | 111.54 | (104.87, 118.20) | 32.82 | <0.001* |
| MoralityCondition [Immoral] | 49.51 | (26.11, 72.91) | 4.15 | <0.001* |
| t1^a^ immoral | 12.25 | (10.68, 13.83) | 15.25 | <0.001* |
| t1^a^ moral | -1.66 | (-2.29, -1.03) | -5.18 | <0.001* |
| t2^b^ immoral | -5.00 | (-5.48, -4.51) | -20.08 | <0.001* |
| t3^c^ immoral | -0.51 | (-0.89, -0.12) | -2.59 | 0.01* |
| Random Effects |  |  |  |  |
| σ^2^ | 20072.72 |  |  |  |
| τ_00_ _Markertrial_ | 251.52 |  |  |  |
| τ_00_ _sjCode_ | 444.25 |  |  |  |
| τ_11_ _sjCode.MoralityConditionImmoral_ | 8509.90 |  |  |  |
| ρ_01_ _sjCode_ | 0.47 |  |  |  |
| ICC | 0.23 |  |  |  |
| Deviance | 2384129.40 |  |  |  |
| log-Likelihood | -1192064.70 |  |  |  |

*Note*. Best model for the analysis of corrugator activity (*N_sjCode_* = 60, *N_Markertrial_* = 64); CI = confidence interval; * significant *p*-value.

^a^ t1 = linear time trend, centered, ^b^ t2 = quadratic time trend, centered, ^c^ t3 = cubic time trend, centered.

Table A2

State Adjective and Affect Reason Linear Mixed Models Analysis Summary.

|  | State Adjective Segment  (M17, *df* = 56084) | | | |  | Affect Reason Segment  (M22, *df* = 93484) | | | |
| --- | --- | --- | --- | --- | --- | --- | --- | --- | --- |
| Predictors | Estimates | (95% CI) | *z* | *p* |  | Estimates | (95% CI) | *z* | *p* |
| (Intercept-Moral Positive) | 110.74 | (99.48, 122.00) | 19.27 | <0.001* |  | 104.72 | (94.63, 114.82) | 20.33 | <0.001* |
| MoralityCondition [Immoral] | 6.48 | (4.37, 8.60) | 6.00 | <0.001* |  | 13.56 | (11.64, 15.48) | 13.84 | <0.001* |
| StateAdjective Condition [Negative] | 22.68 | (19.97, 25.38) | 16.44 | <0.001* |  | 38.99 | (36.54, 41.43) | 31.24 | <0.001* |
| t1^a^ moral negative | 8.25 | (-3.41, 19.92) | 1.39 | 0.17 |  | 6.80 | (-0.28, 13.89) | 1.88 | 0.06 |
| t1^a^ moral positive | -5.47 | (-8.94, -2.00) | -3.09 | 0.002* |  | -3.82 | (-5.71, -1.94) | -3.98 | <0.001* |
| t2^b^ immoral negative | -11.67 | (-35.01, 11.68) | -0.98 | 0.33 |  | -14.56 | (-17.47, -11.64) | -9.78 | <0.001* |
| t2^b^ moral negative | -34.89 | (-43.89, -25.89) | -7.60 | <0.001* |  |  |  |  |  |
| t3^c^ moral negative |  |  |  |  |  | -5.03 | (-11.31, 1.24) | -1.57 | 0.12 |
| MoralityCondition [Immoral] x  StateAdjective Condition [Negative] | -11.36 | (-15.18, -7.54) | -5.83 | <0.001* |  | -35.14 | (-38.25, -32.03) | -22.17 | <0.001* |
| Random Effects |  |  |  |  |  |  |  |  |  |
| σ^2^ | 8159.42 |  |  |  |  | 11172.06 |  |  |  |
| τ_00_ _Markertrial_ | 112.07 |  |  |  |  | 154.63 |  |  |  |
| τ_00_ _sjCode_ | 1840.47 |  |  |  |  | 1417.61 |  |  |  |
| τ_11_ _sjCode.t1_moral_negative_ | 1936.11 |  |  |  |  | 434.86 |  |  |  |
| τ_11_ _sjCode.t2_immoral_negative_ | 7239.30 |  |  |  |  |  |  |  |  |
| τ_11_ _sjCode.t3_moral_negative_ |  |  |  |  |  | 278.58 |  |  |  |
| ρ_01_ _sjCode.t1_moral_negative_ | 0.16 |  |  |  |  | 0.56 |  |  |  |
| ρ_01_ _jCode.t2_immoral_negative_ | -0.33 |  |  |  |  |  |  |  |  |
| ρ_01_ _sjCode.t3_moral_negative_ |  |  |  |  |  | -0.76 |  |  |  |
| ICC | 0.20 |  |  |  |  | 0.12 |  |  |  |
| Deviance | 665242.55 |  |  |  |  | 1137450.49 |  |  |  |
| log-Likelihood | -332621.28 |  |  |  |  | -568725.25 |  |  |  |

*Note*. Best models for the analysis of corrugator activity (*N_sjCode_* = 60, *N_Markertrial_* = 64); CI = confidence interval; * significant *p*-value.

^a^ t1 = linear time trend, centered, ^b^ t2 = quadratic time trend, centered, ^c^ t3 = cubic time trend, centered.

Table A3

Neutral Segment Linear Mixed Models Analysis Summary.

|  | Neutral Segment  (M13, *df* = 93484) | | | |
| --- | --- | --- | --- | --- |
| Predictors | Estimates | (95% CI) | *z* | *p* |
| (Intercept-Moral Positive) | 108.60 | (98.31, 118.89) | 20.68 | <0.001* |
| MoralityCondition [Immoral] | 7.89 | (5.84, 9.94) | 7.54 | <0.001* |
| StateAdjectiveCondition [Negative] | 19.86 | (18.25, 21.47) | 24.23 | <0.001* |
| t1^a^ immoral positive | -3.39 | (-7.87, 1.10) | -1.48 | 0.14 |
| t2^b^ immoral positive | 2.66 | (0.21, 5.10) | 2.13 | 0.03* |
| MoralityCondition [Immoral] x  StateAdjectiveCondition [Negative] | -10.40 | (-13.00, -7.80) | -7.84 | <0.001* |
| Random Effects |  |  |  |  |
| σ^2^ | 7824.57 |  |  |  |
| τ_00_ _Markertrial_ | 124.65 |  |  |  |
| τ_00_ _sjCode_ | 1516.98 |  |  |  |
| τ_11_ _sjCode.t1_immoral_positive_ | 275.49 |  |  |  |
| ρ_01_ _sjCode.t1_immoral_positive_ | -0.86 |  |  |  |
| ICC | 0.18 |  |  |  |
| Deviance | 1104178.80 |  |  |  |
| log-Likelihood | -552089.40 |  |  |  |

*Note*. Best model for the analysis of corrugator activity (*N_sjCode_* = 60, *N_Markertrial_* = 64); CI = confidence interval; * significant *p*-value.

^a^ t1 = linear time trend, centered, ^b^ t2 = quadratic time trend, centered.

Table A4

State Adjective Segment Linear Mixed Models Justice Analysis Summary.

|  | State Adjective Segment  (M17_full, *df* = 56080) | | | |
| --- | --- | --- | --- | --- |
| *Predictors* | *Estimate* | *(95% CI)* | *z* | *p* |
| (Intercept-Moral Positive) | 109.97 | (91.67, 128.26) | 11.78 | <0.001* |
| MoralityCondition [Immoral] | 0.31 | (-3.47, 4.08) | 0.16 | 0.87 |
| StateAdjectiveCondition [Negative] | 12.69 | (8.52, 16.85) | 5.97 | <0.001* |
| Justice score | 1.31 | (-22.56, 25.18) | 0.11 | 0.92 |
| t1^a^ moral negative | 8.44 | (-3.42, 20.29) | 1.39 | 0.17 |
| t1^a^ moral positive | -5.51 | (-9.04, -1.99) | -3.07 | 0.002* |
| t2^b^ immoral negative | -11.76 | (-35.46, 11.93) | -0.97 | 0.33 |
| t2^b^ moral negative | -35.33 | (-44.47, -26.19) | -7.58 | <0.001* |
| MoralityCondition [Immoral] x  StateAdjectiveCondition [Negative] | 0.76 | (-5.52, 7.05) | 0.24 | 0.81 |
| MoralityCondition [Immoral] x  Justice score | 10.66 | (5.46, 15.85) | 4.02 | <0.001* |
| StateAdjectiveCondition [Negative] x Justice score | 17.66 | (12.45, 22.86) | 6.65 | <0.001* |
| MoralityCondition [Immoral] x  StateAdjectiveCondition [Negative] x Justice score | -20.93 | (-29.18, -12.68) | -4.97 | <0.001* |
| Random Effects |  |  |  |  |
| σ^2^ | 8272.36 |  |  |  |
| τ_00_ _Markertrial_ | 117.18 |  |  |  |
| τ_00_ _sjCode_ | 1821.48 |  |  |  |
| τ_11_ _sjCode.t1_moral_negative_ | 1967.61 |  |  |  |
| τ_11_ _sjCode.t2_immoral_negative_ | 7330.35 |  |  |  |
| ρ_01_ _sjCode.t1_moral_negative_ | 0.17 |  |  |  |
| ρ_01_ _sjCode.t2_immoral_negative_ | -0.32 |  |  |  |
| ICC | 0.20 |  |  |  |
| Deviance | 655153.66 |  |  |  |
| log-Likelihood | -327576.83 |  |  |  |

*Note*. Best model for the analysis of corrugator activity (*N_sjCode_* = 59, *N_Markertrial_* = 64); CI = confidence interval; * significant *p*-value.
^a^ t1 = linear time trend, centered, ^b^ t2 = quadratic time trend, centered.

Table A5

Pairwise comparison between corrugator activity at the State Adjective segment for Negative and Positive State Adjectives at different levels of the Justice score.

| **EMM Negative** | **EMM Positive** | **Justice score** | **Morality Condition** | **Estimate**  **Negative-Positive** | **Lower CI** | **Upper CI** | ***df*** | ***t*** | ***p_Sidak_*** |
| --- | --- | --- | --- | --- | --- | --- | --- | --- | --- |
| 110.97 | 107.11 | -0.5 | Moral | 3.86 | -2.50 | 10.22 | 55012.10 | 1.19 | 0.235 |
| 117.17 | 102.09 | -0.5 | Immoral | 15.09 | 7.54 | 22.64 | 12663.08 | 3.92 | <0.001* |
| 120.45 | 107.76 | 0.0 | Moral | 12.69 | 8.52 | 16.85 | 55027.71 | 5.97 | <0.001* |
| 121.52 | 108.07 | 0.0 | Immoral | 13.45 | 8.73 | 18.17 | 18732.07 | 5.58 | <0.001* |
| 129.93 | 108.42 | 0.5 | Moral | 21.51 | 18.72 | 24.31 | 54974.93 | 15.07 | <0.001* |
| 125.86 | 114.05 | 0.5 | Immoral | 11.81 | 8.99 | 14.63 | 54634.75 | 8.21 | <0.001* |
| 139.41 | 109.07 | 1.0 | Moral | 30.34 | 26.90 | 33.79 | 55019.29 | 17.27 | <0.001* |
| 130.20 | 120.03 | 1.0 | Immoral | 10.17 | 6.44 | 13.91 | 29102.91 | 5.34 | <0.001* |
| 148.89 | 109.72 | 1.5 | Moral | 39.17 | 33.75 | 44.60 | 55023.32 | 14.15 | <0.001* |
| 134.55 | 126.01 | 1.5 | Immoral | 8.53 | 2.18 | 14.88 | 14147.81 | 2.63 | 0.008* |

*Note.* EMM = Estimated Marginal Mean, CI = Confidence Interval, * significant *p*-value.

Table A6

Affect Reason Segment Linear Mixed Models Justice Analysis Summary.

|  | Affect Reason Segment  (M22_alt, *df* = 91958) | | | |
| --- | --- | --- | --- | --- |
| *Predictors* | *Estimate* | *(95% CI)* | *z* | *p* |
| (Intercept-Moral Positive) | 102.90 | (85.73, 120.06) | 11.75 | <0.001* |
| MoralityCondition [Immoral] | 1.50 | (-1.92, 4.92) | 0.86 | 0.39 |
| StateAdjectiveCondition [Negative] | 22.56 | (18.80, 26.33) | 11.75 | <0.001* |
| Justice score | 2.91 | (-20.17, 26.00) | 0.25 | 0.81 |
| t1^a^ moral negative | 6.77 | (1.45, 12.10) | 2.49 | 0.013* |
| t1^a^ moral positive | -3.87 | (-5.78, -1.96) | -3.97 | <0.001* |
| t2^b^ moral negative | -14.79 | (-17.74, -11.83) | -9.81 | <0.001* |
| t3^c^ moral negative | -4.86 | (-9.55, -0.16) | -2.03 | 0.043* |
| MoralityCondition [Immoral] x  StateAdjectiveCondition [Negative] | -10.85 | (-15.93, -5.76) | -4.18 | <0.001* |
| MoralityCondition [Immoral] x  Justice score | 20.75 | (16.05, 25.45) | 8.66 | <0.001* |
| StateAdjectiveCondition [Negative] x Justice score | 28.72 | (24.01, 33.42) | 11.96 | <0.001* |
| MoralityCondition [Immoral] x  StateAdjectiveCondition [Negative] x Justice score | -42.13 | (-48.80, -35.47) | -12.39 | <0.001* |
| Random Effects |  |  |  |  |
| σ^2^ | 11284.21 |  |  |  |
| τ_00_ _Markertrial_ | 155.56 |  |  |  |
| τ_00_ _sjCode_ | 1386.22 |  |  |  |
| τ_11_ _sjCode.t1_moral_negative_ | 84.37 |  |  |  |
| ρ_01_ _sjCode.t1_moral_negative_ | -0.01 |  |  |  |
| ICC | 0.12 |  |  |  |
| Deviance | 119802.81 |  |  |  |
| log-Likelihood | -559901.41 |  |  |  |

*Note*. Best model for the analysis of corrugator activity (*N_sjCode_* = 59, *N_Markertrial_* = 64); CI = confidence interval; * significant *p*-value.

^a^ t1 = linear time trend, centered, ^b^ t2 = quadratic time trend, centered, ^c^ t3 = cubic time trend, centered.

Table A7

Pairwise comparison between corrugator activity at the Affect Reason segment for Negative and Positive State Adjectives at different levels of the Justice score.

| **EMM Negative** | **EMM Positive** | **Justice score** | **Morality Condition** | **Estimate**  **Negative-Positive** | **Lower CI** | **Upper CI** | ***df*** | ***t*** | ***p_Sidak_*** |
| --- | --- | --- | --- | --- | --- | --- | --- | --- | --- |
| 107.72 | 99.51 | -0.5 | Moral | 8.20 | 2.46 | 13.95 | 91850.29 | 2.80 | 0.005* |
| 109.06 | 90.64 | -0.5 | Immoral | 18.43 | 12.89 | 23.96 | 91841.94 | 6.53 | <0.001* |
| 123.53 | 100.97 | 0.0 | Moral | 22.56 | 18.80 | 26.33 | 91857.55 | 11.75 | <0.001* |
| 114.19 | 102.47 | 0.0 | Immoral | 11.72 | 8.28 | 15.15 | 91854.04 | 6.69 | <0.001* |
| 139.35 | 102.43 | 0.5 | Moral | 36.92 | 34.39 | 39.45 | 91821.82 | 28.64 | <0.001* |
| 119.31 | 114.30 | 0.5 | Immoral | 5.01 | 3.01 | 7.01 | 91831.13 | 4.90 | <0.001* |
| 155.16 | 103.88 | 1.0 | Moral | 51.28 | 48.17 | 54.39 | 91849.83 | 32.32 | <0.001* |
| 124.43 | 126.13 | 1.0 | Immoral | -1.70 | -4.40 | 1.00 | 91856.73 | -1.24 | 0.22 |
| 170.98 | 105.34 | 1.5 | Moral | 65.64 | 60.74 | 70.54 | 91855.71 | 26.25 | <0.001* |
| 129.56 | 137.97 | 1.5 | Immoral | -8.41 | -13.06 | -3.76 | 91849.36 | -3.55 | 0.001* |

*Note*. EMM = Estimated Marginal Mean, CI = Confidence Interval, * significant p-value

Table A8

Affect Story Fairness Ratings and Justice Scores Linear Mixed Model Analysis Summary.

|  | Fairness Ratings  (M1, *df* = 3677) | | | | Fairness Ratings  (M1_full, *df* = 3673) | | | |
| --- | --- | --- | --- | --- | --- | --- | --- | --- |
| *Predictors* | *Estimate* | *(95% CI)* | *z* | *p* | *Estimate* | *(95% CI)* | *z* | *p* |
| (Intercept-Moral Positive) | 2.06 | (1.97, 2.16) | 42.42 | <0.001* | 1.95 | (1.81, 2.09) | 27.03 | <0.001* |
| MoralityCondition [Immoral] | -3.39 | (-3.50, -3.28) | -60.08 | <0.001* | -3.01 | (-3.19, -2.84) | -34.28 | <0.001* |
| StateAdjectiveCondition [Negative] | -3.50 | (-3.61, -3.39) | -62.11 | <0.001* | -3.25 | (-3.42, -3.07) | -36.99 | <0.001* |
| Justice score |  |  |  |  | 0.19 | (0.01, 0.37) | 2.06 | 0.039* |
| MoralityCondition [Immoral] x  StateAdjectiveCondition [Negative] | 6.44 | (6.29, 6.60) | 80.85 | <0.001* | 5.67 | (5.43, 5.92) | 45.57 | <0.001* |
| MoralityCondition [Immoral] x  Justice score |  |  |  |  | -0.65 | (-0.88, -0.42) | -5.57 | <0.001* |
| StateAdjectiveCondition [Negative] x Justice score |  |  |  |  | -0.44 | (-0.67, -0.21) | -3.72 | <0.001* |
| MoralityCondition [Immoral] x  StateAdjectiveCondition [Negative] x Justice score |  |  |  |  | 1.33 | (1.00, 1.65) | 7.97 | <0.001* |
| Random Effects |  |  |  |  |  |  |  |  |
| σ^2^ | 1.46 |  |  |  | 1.43 |  |  |  |
| τ_00_ _Markertrial_ | 0.02 |  |  |  | 0.02 |  |  |  |
| τ_00_ _sjCode_ | 0.02 |  |  |  | 0.02 |  |  |  |
| ICC | 0.03 |  |  |  | 0.03 |  |  |  |
| Deviance | 11932.39 |  |  |  | 11861.93 |  |  |  |
| log-Likelihood | -5966.19 |  |  |  | -5930.96 |  |  |  |

*Note*. Models for the analysis of Story Fairness ratings (*N_sjCode_* = 59, *N_Markertrial_* = 64); CI = confidence interval; * significant *p*-value.

Table A9

Pairwise comparison between Negative and Positive State Adjectives for Story Fairness ratings at different levels of the Justice score.

| **EMM Negative** | **EMM Positive** | **Justice score** | **Morality Condition** | **Estimate**  **Negative-Positive** | **Lower CI** | **Upper CI** | ***df*** | ***t*** | ***p_Sidak_*** |
| --- | --- | --- | --- | --- | --- | --- | --- | --- | --- |
| -1.18 | 1.85 | -0.5 | Moral | -3.03 | -3.30 | -2.76 | 3619.10 | -21.94 | <.001* |
| 1.15 | -0.83 | -0.5 | Immoral | 1.98 | 1.71 | 2.25 | 3618.45 | 14.36 | <.001* |
| -1.30 | 1.95 | 0.0 | Moral | -3.25 | -3.42 | -3.07 | 3609.93 | -36.99 | <.001* |
| 1.37 | -1.06 | 0.0 | Immoral | 2.43 | 2.26 | 2.60 | 3608.87 | 27.66 | <.001* |
| -1.42 | 2.05 | 0.5 | Moral | -3.46 | -3.58 | -3.35 | 3571.35 | -61.25 | <.001* |
| 1.58 | -1.29 | 0.5 | Immoral | 2.87 | 2.76 | 2.98 | 3569.57 | 50.78 | <.001* |
| -1.54 | 2.14 | 1.0 | Moral | -3.68 | -3.83 | -3.54 | 3598.64 | -49.44 | <.001* |
| 1.79 | -1.53 | 1.0 | Immoral | 3.32 | 3.17 | 3.46 | 3597.83 | 44.54 | <.001* |
| -1.66 | 2.24 | 1.5 | Moral | -3.90 | -4.14 | -3.66 | 3616.01 | -32.12 | <.001* |
| 2.01 | -1.76 | 1.5 | Immoral | 3.76 | 3.53 | 4.00 | 3615.51 | 30.98 | <.001* |

*Note.* EMM = Estimated Marginal Mean, CI = Confidence Interval, * significant *p*-value

Figure A1 Observed Averages of Corrugator Responses in the Neutral Segment.

Average corrugator response at the neutral segment in response to the positive or negative adjective ascribing moral and immoral characters, plotted by time^[1]^. The growth curve model prediction is overlaid.

Figure A2 Whole Trial Observed Average Corrugator Response

Whole trial average corrugator response in 100ms bins, averaged over all trials. The highlighted areas represent the analyzed segments. The corrugator response is given for the four conditions of (im)moral character and positive and negative state adjective. Note that the positive/negative distinction is irrelevant at the character manipulation stage.

1. Interestingly, we do notice a small upwards ‘bump’ in the corrugator patterns for immoral characters directly after the analyzed segment of the adjective (around 0.5-s) for ‘bad Mark happy’, which appears to show a brief phasic increase in corrugator activity as it approaches the pattern for ‘bad Mark frustrated’ more closely than at the adjective (see Figure 3). Although this brief increase may be an indication of a lingering evaluation effect, i.e., ‘it is unfair that bad Mark feels happy’, the patterns still do not flip.
